# Validity and reliability of a semi-quantitative food frequency questionnaire in groups at high risk for cardiovascular diseases

**DOI:** 10.1186/s12937-022-00815-8

**Published:** 2022-10-14

**Authors:** Ni Yan, Nan Li, Wanlu Liu, Xiaoxia Li, Xiuying Liu, Pengju Zhang, Can Liu, Juan Li, Jiangwei Qiu, Yuhong Zhang, Yi Zhao

**Affiliations:** 1 School of Public Health and Management, Ningxia Medical University, Yinchuan, China; 2 Key Laboratory of Environmental Factors and Chronic Disease Control, No.1160, Shengli Street, Xingqing District, Yinchuan, Ningxia China

**Keywords:** Food frequency questionnaire, Reliability, Validity, CVD, Food groups

## Abstract

**Background:**

Diet is a modifiable risk factor for cardiovascular diseases (CVD), but there is still a lack of tools to assess dietary intakes of this high-risk population in Ningxia, China.

**Objective:**

We aim to evaluate the validity and reliability of the semi-quantitative food frequency questionnaire (SFFQ) in the groups in Ningxia using a 24-hour dietary recall method.

**Method:**

Two hundred five participants were included in the analysis. The two FFQs were 6 months apart, and during this time two 24-hour dietary recalls (24HDRs) were completed. Statistical methods were compared using inter-class correlation coefficient, unadjusted, energy-adjusted, de-attenuated correlation coefficient, quartile classification, weighted K values, and 95% limits of agreement (LOA).

**Results:**

The inter-class correlation coefficients between FFQ1 and FFQ2 ranged from 0.25 to 0.73. The number of subjects classified as identical or adjacent was 72.2 to 85.9%. The crude correlation coefficient between FFQs and 24HDRs was 0.30 ~ 0.81, the energy-adjusted correlation coefficient was 0.16 ~ 0.83, and the de-attenuated correlation coefficient was 0.19 ~ 0.98. Weighted k statistics and Bland-Altman plots showed acceptable agreement between FFQs and 24HDRs.

**Conclusion:**

The FFQ developed for the population at high risk of cardiovascular and cerebrovascular diseases in areas of Ningxia can be used to measure the dietary intake of nutrients and food groups reliably and validly.

**Supplementary Information:**

The online version contains supplementary material available at 10.1186/s12937-022-00815-8.

## Introduction

Cardiovascular disease (CVD) is one of the leading causes of death and disability globally. In the past 10 years, the global mortality rate of CVD has increased by 12.5% [1]. CVD is characterized by high morbidity, disability, mortality and recurrence rates [2]. According to the Global Burden of Diseases 2017, stroke and ischemic heart disease are the top two causes of death in China. Accounting for stroke and ischemic heart disease of deaths annually is 149 and 124 per 100,000 population [3]. Dietary risk factors have been found to influence the occurrence, progression and mortality of CVD [2, 4, 5]. Reasonable dietary intakes including Mediterranean Diet supplemented with extra-virgin olive oil and Mediterranean Diet supplemented with nuts can reduce the occurrence of CVD by 50% [6]. Many epidemiological studies have also shown that appropriate dietary habits play a very important role in the protection and prevention of CVD and adapting appropriate dietary patterns such as diets high in monounsaturated and polyunsaturated acids that favor metabolic markers may prevent CVD [7]. On the contrary, malnutrition may lead to an increase in morbidity and mortality. Considering that nutrition is an important modifiable risk factor for people at high risk of CVD, it is important to understand the current dietary intake [8]. Therefore need to have appropriate assessment tools for dietary patterns [9].

In nutritional epidemiology, there have been numerous tools to assess dietary intake and each method has its own advantages and limitations [10–12]. The most commonly used assessment tools are the 24-hour dietary recall and dietary records containing weighing foods are also used to measure daily dietary intake, but these methods are expensive, time-consuming, and not suitable for most large-scale studies [13]. Besides, short-term recalls and diet records are not representative of usual dietary intake. Therefore, it is not sufficient to assess dietary intakes over time. Food Frequency Questionnaire (FFQ) is considered to be a low-cost diet assessment method suitable for a large sample and is often used to research the relationship between dietary factors and diseases in various epidemiological studies [14, 15]. Due to its long reference period and pre-specified food list, the collected dietary intake information has limitations to accurately measure the dietary intakes [16]. In other words, the nutritional content of FFQ is mainly affected by systematic errors and requires a careful evaluation when assessing diet-disease relationships [17, 18]. Also, the differences in demographic, socio-economic, cultural, and other points also influence the food intake of each group [19, 20]. Therefore, it is necessary to verify the validity and reliability of the FFQ in each group to evaluate the accuracy and precision of dietary intake.

A recent study evaluated the effectiveness of an SFFQ in a group with CVD [18]. The results showed that due to the short time span of SFFQ, there were large seasonal differences in nutrient estimates. Thus, there are some limitations in the research. In addition, the result cannot be transferred to other groups in different dietary regions, and each region should develop localized SFFQ based on its specific dietary habits and traditions rather than using a uniform SFFQ. The mortality rate of CVD in Ningxia residents showed an increasing trend from 2012 to 2016 and was mainly over 60 years old [21]. At the same time, the unreasonable dietary patterns of adult residents in Ningxia was manifested by insufficient intake of vegetables and fruits and excessive intake of salt and edible oil, which can increase the risk of related diseases [22]. We were interested in the consumption of polyunsaturated fatty acids in this population. Because the program was a long-term dietary intervention, FFQ was a relatively good dietary survey method.

Therefore, the aim of this study was to assess the validity and reliability of an SFFQ used to assess dietary intakes in groups at high risk for CVD in Ningxia.

## Methods

This study collected two FFQs over6 months. The first FFQ (FFQ1) was collected in December 2019 and the second FFQ (FFQ2) was collected in July 2020. Two discontinuous 24-hour dietary recalls were collected, at the same time as FFQs. All the dietary interviewers were conducted on a random day because pre-trial tests showed there was no significant difference between weekdays and weekends in the diets of residents in the areas.

### Study populations

Participants have been recruited from six villages from Qingtong Xia County of Ningxia province in China and they were same as the participants of project [23]. Based on the study of Willett [10], at least 110 participants were required to examine validity and reliability of dietary surveys. The study recruited 210 participants at high risk of CVD, defined as having a history of CVD. In each village, A history of CVD or a high risk of CVD, defined on the basis of a prior hospitalisation or male aged> 60 years, or female aged> 65 years, and with at least two of the following risk factors: a. Type 2 diabetes requiring treatment with at least two oral anti-hyperglycaemic agents and/or insulin b. Systolic blood pressure > 140 mmHg while on one or more antihypertensive agents c. Current daily smoking. Participants with an intellectual disability, a cognitive disability or with any chronic medical condition which required dietary restriction were excluded. The doctors will inform those participants to prepare relevant supporting documents (medical records and physical evidence of cigarettes and drugs) prior to participating in screening interviews.

The study was reviewed and confirmed by the Ethics Committee of Ningxia Medical University (No. 2020–066).

### The SFFQ

The SFFQ was developed from a pre-trial of 20 people, conducted by household interviews. In the pre-trial, food pictures were used to help recall food types. There were two standardized trained staff on site. The SFFQ was formulated by combining the types of foods that were based on the 3 days 24-hour dietary recalls survey in local household. The questionnaire concluded 173 types of typical foods from areas of Ningxia, including foods unique to northwest China and accounting for about 95% of the most commonly used foods in Ningxia. Participants had the option to type name of foods if not in the SFFQ. These food items were allocated to 15 food groups such as: cereals, beans and soy products, potatoes, milk and dairy products, meat and meat products, eggs, aquatic products, vegetables, fruit, the fungus mushrooms, oils and fats, alcohol, beverages, snacks and condiments. For each food item, four categories were provided for frequencies (daily, weekly, monthly, annually or never) and the amount of consumption over the past 6 months was reported using the common weight unit in China (1 liang =50 g) [24]. The monthly consumption of the whole family was used for estimation of oil and condiments consumption. The SFFQ was designed to survey the dietary intakes of participants over the past 6 months and was administered by two staff with standard training due to the low educational level of the participants.

### The 24-hour dietary recall

All recall interviews were conducted in the subjects’ homes to estimate commonly used home measurements more accurately and to limit the number of missing subjects. Participants were asked to recall all the foods or beverages they had eaten in the previous 24 hours and estimate the portion size. Common household measurements (the size of bowls and plates) are used to help estimate portions. Other dietary information includes recipe ingredients, the measures of cooking and the time and place of they had eaten (e.g. home or outside). Mixed foods in the 24-h dietary recall are converted to their ingredients to measure.

### Calculation of nutrient intake

The data obtained from the FFQ was imported with EpiData software by two trained staff. The data from the 24-hour dietary recalls were inputted into the Nutrition Calculator (v2.7.5(k), Institute for Nutrition and Food Security, Chinese Center for Disease Control and Prevention). The daily nutrient intake of participants was calculated using the China Food Composition Database. The nutritional values of each food were calculated by matching the food names in the database. If there is one food not listed in the database, the nutritional values were calculated using foods that contain similar ingredients. The data of food intake and nutrient intake output by the dietary software were imported into Microsoft Excel for statistical analysis.

### Statistical analysis

To measure the consistency of the first and second FFQ received by the same subject at different times, Spearman’s rank correlation coefficient (r) was calculated to compare the energy, nutrients and food intakes between two FFQ administrations. For reliability analysis, we used the inter-class correlation coefficient (ICC) and 95% CI for each point. Weighted kappa (k) statistic, and misclassification (quartiles method) analyses also were used to assess the reliability between FFQ1 and FFQ2. Among them, k values above 0.80 indicate very good agreement, 0.61 to 0.80 indicate substantial (good) agreement, 0.41 to 0.60 indicate moderate agreement, 0.21 to 0.40 indicate good agreement, and 0 to 0.20 indicate slight (poor) agreement [25].

The overall raw data were natural-log(ln) transformed to improve the normality of food groups and nutrients. Spearman’s rank correlation coefficient (r) of natural-log(ln) transformed values were calculated to evaluate the validity of FFQ and 24-hour dietary recalls. The FFQ and 24-hour dietary recall data were the mean of the two times. Energy-adjusted nutrients intakes estimates were obtained by the residual method [10]. All validity coefficients were attenuated due to random errors in the 24-hour dietary recalls. This formula from Willet was used to calculate the de-attenuated correlation to eliminate within-person variability in 24-hour recalls [10]: ${r}_t={r}_0\sqrt{1+r/n}$, where r_0_ is the observed correlation between FFQs and 24-hour recalls, and r is the rate of variation within- and between-person measured during two 24-hour recalls, and n is the number of days of dietary recall (*n* = 2). Bland-Altman plots were used to test the consistency of the two dietary assessment methods between different intakes. As suggested by Bland&Altman [26], the natural-log(ln) transformed was performed to narrow the 95% limits of agreement (LOA).

IBM SPSS Statistics Version23.0 was used for all data analysis. All *p* values were double-tailed, and the *p* values less than 0.05 were considered evidence of a statistically significant correlation.

## Results

Among the 210 selected participants, 205 agreed to take part in the study and completed the survey (response rate = 97.6%). The main reasons for not participating in the study included refusal, absence during the investigation period, poor health and death. Table 1 lists the sociodemographic and anthropometric characteristics of the study population. The mean age of the subjects was 65.3 years old (male 46.3%), the mean height was 160.4 cm, the mean weight was 68.1 kg, and the mean body mass index was 26.4 kg /m^2^.

**Table 1:** Characteristics and anthropometric measurements of the participants included (*N* = 205)

Characteristics^a^	Male(*n* = 110)	Female(*n* = 95)	All(*n* = 205)
Age at recruitment (years)	65.0 ± 7.7	65.6 ± 6.8	65.3 ± 7.3
Age range (years)	34–80	42–77	34–80
Degree of education (number (%))			
Primary school or below	71 (64.5)	79 (83.2)	150 (73.2)
Middle school	29 (26.4)	8 (8.4)	37 (18.0)
High school and above	10 (9.1)	8 (8.4)	18 (8.8)
Height (cm)	165.8 ± 5.8	154.0 ± 4.7	160.4 ± 7.9
Weight (kg)	72.6 ± 10.5	62.8 ± 9.2	68.1 ± 11.0
BMI (kg/m2)^b^	26.3 ± 3.2	26.5 ± 3.6	26.4 ± 3.4
Waist (cm)	92.8 ± 9.8	89.4 ± 9.1	91.3 ± 9.6
Hip (cm)	98.1 ± 5.2	95.2 ± 5.4	96.8 ± 5.5
WHR^b^	0.94 ± 0.06	0.94 ± 0.06	0.94 ± 0.06

^a^Continuous normally distributed variables are expressed as mean and SD

^b^*BMI* body mass index, *WHR* Waist to hip ratio

The median intakes of total energy, nutrients, and food groups derived from the two FFQ and the percentage of differences are presented in Table 2. The median intakes for all nutrients and food groups (except fruit) evaluated with FFQ2 were higher than or equal to the median intakes with FFQ1, with differences between 0 and 52.4%. The largest differences between the median intakes of the two FFQ were 36.0% for nutrients (cholesterol) and 52.4% for the food group (eggs).

**Table 2:** Comparison of nutrients intakes between two FFQs for 205 participants (median and 25th - 75th percentiles)

Food groups or nutrients	FFQ1		FFQ2		Percentages of median difference
	Median	25–75th percentile	Median	25–75th percentile	
Food groups(g/d)					
Tuber crops	47.0	21.6–94.0	47.0	25.9–70.5	0.0^b^
Meats	29.5	14.7–63.7	37.5	19.1–69.1	21.2^b^
Eggs	8.8	1.3–18.5	18.5	3.5–37.0	52.4^a^
Grains	416.5	307.3–547.0	443.5	328.9–540.4	6.1
Oil	33.3	25.0–44.4	33.3	25.0–44.4	0.0
Vegetables	387.0	230.3–522.0	510.0	383.3–637.0	24.1^a^
Fruits	4.2	0.4–14.0	4.0	0.1–11.2	−3.5^b^
Aquatic products	42.3	20.0–100.0	64.2	40–101.4	34.1
Legumes and products	42.9	20.0–65.8	45.5	23.2–71.7	5.7
Dairy and products	16.8	0.0–84.0	40.0	0.0–87.6	58.0
Salty agent	10.0	7.4–13.1	10.0	7.4–13.3	0.0
Spicy agent	16.7	8.3–20.8	16.7	8.3–25.0	0.0
Sour agent	1.0	0.3–2.5	1.0	0.3–2.1	0.0
Nutrients					
Energy(kcal/d)	1633.1	1329.9–2064.2	1912.0	1483.4–2287.8	14.6^a^
Carbohydrate(g/d)	61.0	44.9–79.0	70.4	57.6–88.8	13.4^a^
Protein(g/d)	49.2	39.0–65.2	60.2	44.1–72.7	18.2^a^
Dietary fiber(g/d)	11.8	8.0–15.1	14.9	11.6–17.9	21.2^a^
Cholesterol(mg/d)	104.9	57.2–178.0	163.9	84.5–257.1	36.0^a^
Fat(g/d)	222.4	171.2–289.7	248.1	187.3–310.9	10.4^a^
SFA(g/d)	9.0	6.2–12.2	10.5	7.7–13.9	14.6^a^
MUFA(g/d)	21.3	16.0–29.8	25.8	20.8–35.1	17.4^b^
PUFA(g/d)	19.7	14.0–25.8	21.1	16.1–27.6	6.9^a^
Vitamin A(μgRE/d)	263.4	174.5–380.7	350.6	270.0–447.7	24.9
Vitamin D(μg/d)	0.8	0.4–1.7	1.1	0.5–1.9	23.3
Vitamin E(mg/d)	82.6	22.3–146.7	88.2	30.2–151.2	6.3
Thiamin(mg/d)	0.8	0.6–1.0	0.9	0.7–1.1	9.8^a^
Riboflavin(mg/d)	0.8	0.6–1.0	0.9	0.7–1.1	14.1^a^
Pyridoxine(mg/d)	0.3	0.2–0.5	0.4	0.3–0.5	9.2^a^
Vitamin C(mg/d)	100.3	60.5–140.4	133.7	98.9–167.5	25.0^a^
Folate(μg/d)	159.1	103.8–220.6	208.7	160.3–267.8	23.8^a^
Niacin(mg/d)	14.5	11.4–18.5	16.5	12.5–20.4	12.0^a^
Calcium(mg/d)	383.7	277.8–499.6	492.0	378.2–605.6	22.0^a^
Phosphorus(mg/d)	843.5	668.6–1080.0	998.2	788.1–1188.0	15.5^a^
Potassium(mg/d)	1783.2	1331.8–2364.8	2251.3	1731.6–2621.4	20.8^a^
Sodium(mg/d)	3804.4	3015.3–4748.1	3843.8	2968.8–4828.0	1.0
Magnesium(mg/d)	306.9	232.3–371.5	361.0	286.0–425.2	15.0^a^
Iron(mg/d)	21.2	16.2–26.1	24.7	18.7–29.5	14.3^a^
Zinc(mg/d)	9.0	7.1–11.4	10.4	8.0–12.6	14.2^a^
Selenium(μg/d)	27.8	22.1–39.1	34.3	25.5–43.3	19.0^a^
Copper(mg/d)	1.5	1.1–1.9	1.7	1.3–2.1	12.2^a^
Iodine(μg/d)	31.0	20.8–58.5	42.1	28.0–68.4	26.4

a and b mean *P* value for test of difference < 0.001 and < 0.05 respectively

Notes: Vegetables including fresh vegetables and pickled vegetables; Aquatic products including fish, shrimp and seaweeds; *SFA* saturated fatty acid, *PUFA* polyunsaturated fatty acids, *MUFA* monounsaturated fatty acids

The ICCs of food groups ranged from 0.34 to 0.73 and nutrients ranged from 0.25 to 0.72 (Table 3). The energy-adjusted ICCs of food groups ranged from 0.26 to 0.73 and nutrients ranged from 0.26 to 0.71. When the food groups and nutrient intakes were divided into quartiles, the agreement rates of FFQ1 and FFQ2 in the same and adjacent quartile were 72.2 to 85.9%. Except for vitamin A(10.2%), the misclassification for all nutrients and food groups as extreme quartile was rare (< 10%). Most the weighted k statistics were moderate conformity, ranging from 0.40 to 0.54. Weighted k statistics for the five food groups and seven nutrients were general conformity, ranging from 0.29 to 0.39. Only vegetables showed poor consistency, with a low weighted k statistic (0.19).

**Table 3:** Reliability of food groups and nutrients intakes between FFQ1 and FFQ2

Food groups or nutrients	ICC(95%CI)		Percent of agreement(%)				Weighted k
	Crude	Energy adjusted	Same quartile	Adjacent quartile	One quartile apart	Opposite quartile	
Food groups(g/d)							
Tuber crops	0.65(0.54,0.74)^a^	0.66(0.55,0.74)^a^	43.9	42.0	12.2	1.9	0.52
Meats	0.58(0.45,0.68)^a^	0.53(0.38,0.64)^a^	36.6	41.5	17.6	4.3	0.39
Eggs	0.55(0.40,0.66)^a^	0.61(0.49,0.71)^a^	42.4	37.1	15.1	5.4	0.47
Grains	0.66(0.55,0.74)^a^	0.59(0.46,0.69)^a^	38.5	39.5	19.5	2.5	0.44
Oil	0.73(0.65,0.80)^a^	0.73(0.64,0.79)^a^	39.5	32.7	22.0	5.8	0.38
Vegetables	0.34(0.13,0.50)^b^	0.26(0.03,0.44)^b^	30.2	42.0	18.5	9.3	0.19
Fruits	0.39(0.19,0.53)^a^	0.47(0.30,0.59)^a^	44.9	35.6	15.6	3.9	0.46
Aquatic products	0.59(0.47,0.69)^a^	0.54(0.40,0.65)^a^	46.8	36.1	13.2	3.9	0.50
Legumes and products	0.68(0.58,0.76)^a^	0.59(0.46,0.69)^a^	45.9	38.0	13.7	2.4	0.54
Salty agent	0.57(0.43,0.67)^a^	0.49(0.33,0.61)^a^	34.2	39.5	21.0	5.3	0.32
Spicy agent	0.58(0.45,0.68)^a^	0.60(0.47,0.70)^a^	36.6	39.5	21.5	2.4	0.38
Sour agent	0.64(0.53,0.73)^a^	0.59(0.46,0.69)^a^	44.4	34.6	19.0	2.0	0.36
Nutrients							
Energy(kcal/d)	0.68(0.57,0.75)^a^	0.68(0.57,0.75)^a^	38.1	45.4	13.7	2.8	0.49
Carbohydrate(g/d)	0.65(0.53,0.73)^a^	0.56(0.42,0.66)^a^	38.1	44.9	13.7	3.3	0.48
Protein(g/d)	0.68(0.57,0.75)^a^	0.66(0.56,0.74)^a^	46.3	34.6	15.1	4.0	0.48
Dietary fiber(g/d)	0.58(0.45,0.68)^a^	0.60(0.47,0.69)^a^	35.6	43.9	15.6	4.9	0.4
Cholesterol(mg/d)	0.62(0.50,0.71)^a^	0.67(0.57,0.75)^a^	42.0	40.0	15.6	2.4	0.5
Fat(g/d)	0.65(0.54,0.73)^a^	0.62(0.50,0.71)^a^	36.1	41.0	18.1	4.8	0.37
SFA(g/d)	0.60(0.48,0.70)^a^	0.62(0.50,0.71)^a^	37.6	43.4	15.1	3.9	0.44
MUFA(g/d)	0.62(0.50,0.71)^a^	0.64(0.53,0.73)^a^	40.0	37.6	18.5	3.9	0.41
PUFA(g/d)	0.67(0.57,0.75)^a^	0.61(0.48,0.70)^a^	32.7	43.9	18.5	4.9	0.35
Vitamin A(μgRE/d)	0.27(0.04,0.44)^b^	0.26(0.02,0.44)^b^	37.6	35.1	16.6	10.7	0.33
Vitamin D(μg/d)	0.65(0.54,0.73)^a^	0.62(0.50,0.71)^a^	43.4	39.5	12.7	4.4	0.48
Vitamin E(mg/d)	0.72(0.63,0.79)^a^	0.69(0.60,0.77)^a^	46.3	37.1	13.7	2.9	0.53
Thiamin(mg/d)	0.63(0.51,0.72)^a^	0.61(0.48,0.70)^a^	41.5	38.1	15.6	4.8	0.42
Riboflavin(mg/d)	0.65(0.53,0.73)^a^	0.70(0.60,0.77)^a^	39.0	43.9	12.7	4.4	0.43
Pyridoxine(mg/d)	0.54(0.39,0.65)^a^	0.54(0.40,0.65)^a^	41.5	37.6	16.1	4.8	0.41
Vitamin C(mg/d)	0.25(0.02,0.43)^b^	0.28(0.05,0.45)^b^	36.1	36.1	22.0	5.8	0.29
Folate(μg/d)	0.53(0.38,0.64)^a^	0.50(0.35,0.62)^a^	35.1	42.9	16.1	5.9	0.36
Niacin(mg/d)	0.66(0.55,0.74)^a^	0.56(0.41,0.66)^a^	40.5	42.4	13.2	3.9	0.48
Calcium(mg/d)	0.62(0.50,0.71)^a^	0.64(0.52,0.72)^a^	40.5	37.6	15.6	6.3	0.37
Phosphorus(mg/d)	0.65(0.54,0.74)^a^	0.65(0.54,0.73)^a^	45.4	36.1	14.6	3.9	0.48
Potassium(mg/d)	0.65(0.54,0.73)^a^	0.58(0.44,0.68)^a^	45.4	36.1	13.2	5.3	0.45
Sodium(mg/d)	0.58(0.45,0.68)^a^	0.49(0.32,0.61)^a^	37.1	38.1	18.5	6.3	0.32
Magnesium(mg/d)	0.61(0.49,0.70)^a^	0.56(0.42,0.66)^a^	42.9	37.1	15.6	4.4	0.44
Iron(mg/d)	0.68(0.58,0.76)^a^	0.71(0.61,0.78)^a^	42.9	38.1	15.6	3.4	0.47
Zinc(mg/d)	0.65(0.53,0.73)^a^	0.62(0.50,0.71)^a^	41.0	42.4	11.2	5.4	0.46
Selenium(μg/d)	0.63(0.51,0.72)^a^	0.57(0.44,0.67)^a^	43.4	34.6	17.1	4.9	0.41
Copper(mg/d)	0.58(0.45,0.68)^a^	0.54(0.40,0.65)^a^	44.4	35.6	15.6	4.4	0.45
Iodine(μg/d)	0.65(0.53,0.73)^a^	0.68(0.58,0.76)^a^	42.0	42.9	9.8	5.3	0.48

a and b mean *P* value for ICC < 0.001 and < 0.05 respectively

Notes: FFQ1 first FFQ, FFQ2 FFQ after six months, *ICC* intra-group correlation coefficient, *Weighted K* weighted Kappa value, *SFA* saturated fatty acid, *PUFA* polyunsaturated fatty acids, *MUFA* monounsaturated fatty acids; Vegetables including fresh vegetables and pickled vegetables; Aquatic products including fish, shrimp and seaweeds; Residual method was adopted for energy adjustment

Overall, the median daily intakes of nutrients, assessed by two FFQs average, were substantially higher than the average of the two 24-hour dietary recalls, except for protein, carbohydrates, and vitamin B1 (Table 4). The unadjusted Spearman correlation coefficients for nutrients ranged from 0.18 for vitamin A to 0.81 for vitamin E. After energy adjustment, Spearman correlation coefficients of all nutrients decreased except vitamin E, which increased slightly. Compared with the unadjusted values, niacin (0.59 ~ 0.27) and iron (0.45 ~ 0.14) had the most significant changes. Except for vitamin A, vitamin D, folic acid, and iodine, the energy-adjusted coefficients of the other nutrients were statistically significant (*p* < 0.01 or *p* < 0.05). The average of correlation coefficients improved from 0.32 to 0.39 after energy adjustment, and correction for random within-person variation and between-person variation. The de-attenuated correlations for all the nutrients increased and ranged from 0.07 for folic acid to 0.98 for vitamin E. When nutrients intakes were divided into quartiles, the agreement rates between FFQs and 24-hour dietary recalls of the same and adjacent quartiles were 71.2 to 94.1%. The misclassification for all nutrients as extreme quartile was rare (< 10%), except for vitamin D (12.7%) and iodine (10.2%).

**Table 4:** Validity of nutrients intakes between FFQs and 24HDRs

Nutrients	FFQs		24HDRs		Percentages of median difference	Spearman (r)			Percent of agreement(%)			
	Median	25–75th percentiles	Median	25–75th percentiles		Crude	Energy adjusted	De-attenuated	Same quartile	Adjacent quartile	One quartile apart	Opposite quartile
Energy(kcal/d)	1792.0	1475.3–2164.1	1787.0	1426.3–2146.0	0.3	0.61^a^	–	–	44.9	42.0	10.7	2.4
Carbohydrate(g/d)	235.4	189.4–299.1	272.6	202.1–328.0	−15.8^a^	0.63^a^	0.47^a^	0.55	48.8	37.1	12.2	1.9
Protein(g/d)	54.1	44.6–67.2	55.0	43.0–67.0	−1.7	0.58^a^	0.39^a^	0.45	38.5	45.9	13.7	1.9
Dietary fiber(g/d)	13.1	10.5–16.2	11.1	8.7–13.8	15.5^a^	0.46^a^	0.38^a^	0.47	41.5	38	17.6	2.9
Cholesterol(mg/d)	140.8	77.5–216.3	46.0	12.5–144.0	67.3^a^	0.43^a^	0.36^a^	0.44	35.6	41.5	18	4.9
Fat(g/d)	66.5	54.1–79.4	52.4	39.4–63.5	21.2^a^	0.55^a^	0.53^a^	0.63	43.9	37.6	15.6	2.9
SFA(g/d)	9.8	7.6–12.7	8.1	6.3–10.8	17.3^a^	0.35^a^	0.38^a^	0.46	39.5	42.0	14.1	4.4
MUFA(g/d)	20.9	16.5–26.0	15.8	12.5–21.1	24.4^a^	0.49^a^	0.56^a^	0.69	42.9	42.4	11.7	3.0
PUFA(g/d)	24.3	19.5–31.0	19.8	14.1–25.5	18.5	0.04^a^	0.11	0.13	36.6	49.8	10.7	2.9
Vitamin A(μgRE/d)	300.5	243.0–415.6	155.0	98.5–277.5	48.4^a^	0.18^b^	0.10	0.12	27.3	43.9	19.0	9.8
Vitamin D(μg/d)	1.0	0.6–1.9	0.0	0.0–0.6	100^b^	0.23^b^	0.08	0.10	31.7	35.1	20.5	12.7
Vitamin E(mg/d)	88.9	35.8–145.0	73.6	34.6–109.8	17.2^a^	0.81^a^	0.83^a^	0.98	62.4	31.7	5.4	0.5
Thiamin(mg/d)	0.8	0.7–1.1	1.1	0.8–1.4	−30.4^a^	0.55^a^	0.32^a^	0.37	46.8	38.0	11.2	4.0
Riboflavin(mg/d)	0.9	0.7–1.0	0.5	0.4–0.6	46.7^a^	0.55^a^	0.37^a^	0.45	37.6	44.4	16.6	1.4
Pyridoxine(mg/d)	0.4	0.3–0.5	0.3	0.2–0.4	21.0^a^	0.35^a^	0.22^b^	0.26	37.1	39.0	15.6	8.3
Vitamin C(mg/d)	115.1	90.3–148.8	81.4	54.1–114.7	29.3^a^	0.30^a^	0.16^b^	0.19	34.6	39.5	18.5	7.4
Folate(μg/d)	186.2	147.0–231.6	133.3	105.3–173.2	28.4^a^	0.31^a^	0.06	0.07	32.7	43.9	16.6	6.8
Niacin(mg/d)	16.0	12.4–19.4	10.4	8.3–13.1	34.6^a^	0.59^a^	0.27^a^	0.32	39.5	45.9	13.2	1.4
Calcium(mg/d)	449.0	359.4–535.3	263.0	199.0–326.8	41.4^a^	0.41^a^	0.33^a^	0.39	35.6	36.6	23.4	4.4
Phosphorus(mg/d)	917.1	762.6–1123.0	737.7	585.3–890.8	19.6^a^	0.59^a^	0.40^a^	0.47	46.3	37.6	15.1	1.0
Potassium(mg/d)	2000.4	1634.4–2452.6	1487.6	1181.9–1799.9	25.6^a^	0.48^a^	0.38^a^	0.45	43.4	36.1	18.0	2.5
Sodium(mg/d)	3961.4	3200.3–4623.2	2910.8	2431.4–3556.3	26.5^a^	0.53^a^	0.48^a^	0.59	42.0	38.5	16.6	2.9
Magnesium(mg/d)	320.3	277.5–398.5	258.0	202.0–311.5	19.4^a^	0.57^a^	0.40^a^	0.48	46.8	35.6	15.6	2.0
Iron(mg/d)	22.9	18.6–26.9	9.7	7.8–12.1	57.9^a^	0.45^a^	0.14^b^	0.17	37.6	40.5	19.5	2.4
Zinc(mg/d)	9.7	7.8–11.7	6.1	4.8–7.5	36.7^a^	0.54^a^	0.26^a^	0.31	48.3	34.6	15.6	1.5
Selenium(μg/d)	32.3	25.1–39.0	26.7	20.0–34.6	17.4^a^	0.59^a^	0.41^a^	0.49	42.4	42.9	12.7	2.0
Copper(mg/d)	1.6	1.3–2.0	1.2	0.9–1.5	22.3^a^	0.48^a^	0.33^a^	0.41	35.1	44.4	19.0	1.5
Iodine(μg/d)	40.6	27.2–62.1	21.3	15.7–28.7	47.6^a^	0.22^b^	0.10	0.12	29.8	42.4	17.6	10.2

a and b mean *P* value for test for difference and Spearman < 0.001 and < 0.05 respectively

Notes: *SFA* saturated fatty acid, *PUFA* polyunsaturated fatty acids, *MUFA* monounsaturated fatty acids; Residual method was adopted for energy adjustment

**Fig. 1:**
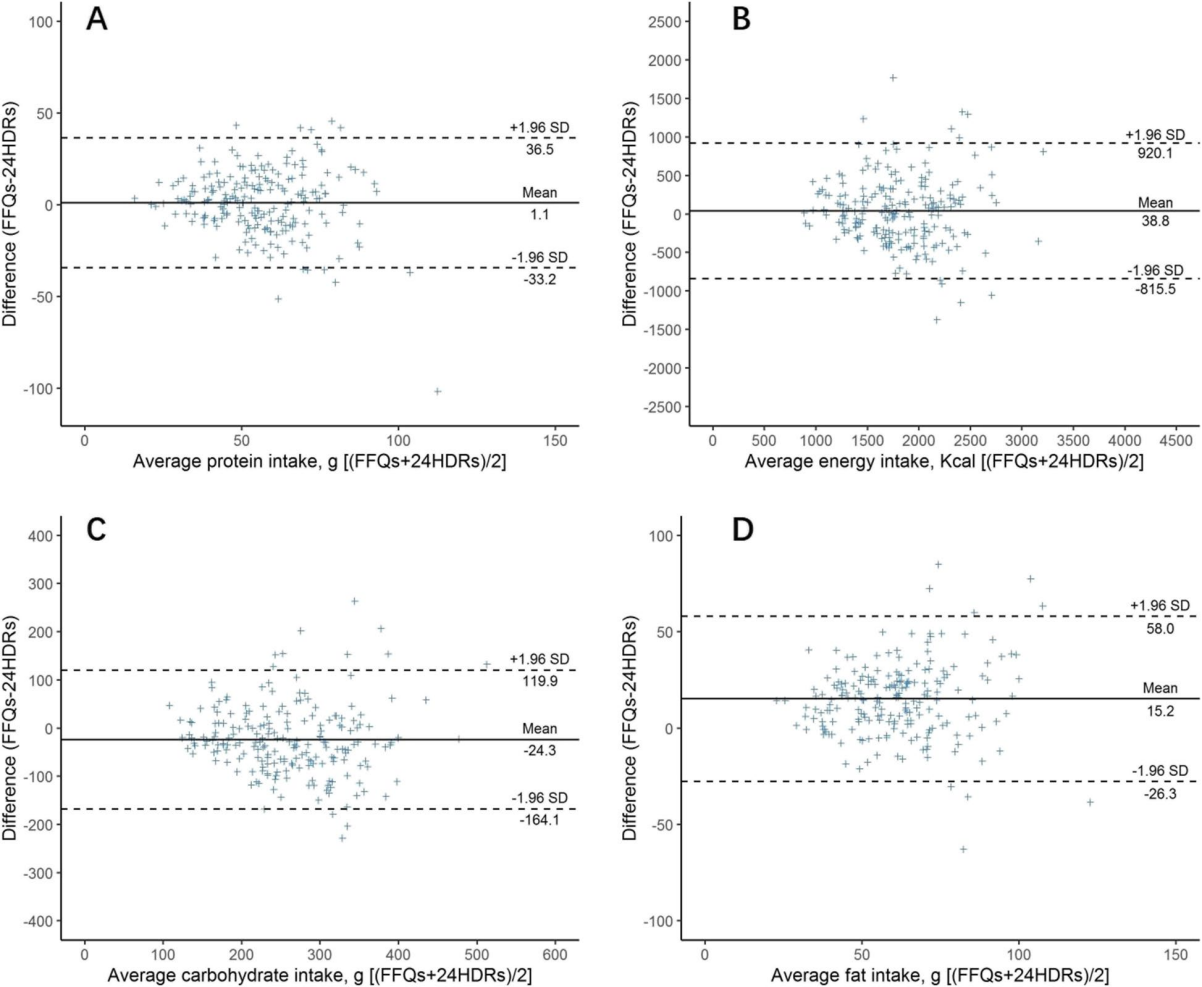
Bland-Altman plots for nutrients between FFQs and 24HDRs to assess the intake of: (**A**) protein, (**B**) energy, (**C**) carbohydrate, and (**D**) fat. The limits of agreement (dotted line) indicates the 95% confidence interval(mean ± 1.96SD)

The results of the Bland-Altman plots showed in Fig. 1 including energy, protein, fat, and carbohydrates (Other nutrients were listed in Annex). According to Fig. 1, most of the points fell within the 95% limits of agreement (LOAS), closer to the middle horizontal line and there was no linear trend between the differences and means for two FFQs and 24-hour dietary recalls.

## Discussion

This study aimed to examine the validity and reliability of SFFQ used to evaluate various food groups and nutrients in dietary intakes assessment of high-risk populations of cardiovascular disease in Ningxia. In this study, two discontinuous 24-hour dietary recalls were collected to evaluate the reliability and validity of the SFFQ at 6-month intervals. We assessed the performance of SFFQ by comparing the food groups and nutrients intakes reported using the instrument with intakes obtained using the 24-hour dietary recalls. Overall, the SFFQ has shown acceptable reliability and validity in evaluating food groups and nutrient intakes in people at high risk for CVD.

In previous studies [27, 28], the intervals between FFQs varied. Reliability is based on the results of diet between two questionnaires, ideally the shorter the interval, the better the reliability [10]. In this case, however, the subjects were more likely to remember and repeat their previous answers. In this study, there was a six-month interval between the two FFQs, which could relatively reduce the above bias. Similarly, for validation studies, it is crucial to select the appropriate reference method to evaluate FFQ. Previous studies have shown that FFQ and 24-hour dietary recalls have been validated in 75% of studies. Although FFQ and 24-hour dietary recall are both retrospective questionnaires, they are prone to the same recall bias, which may lead to overestimation of the correlation coefficient. However, 24-hour dietary recalls may be more appropriate for subjects with lower educational levels [20, 29]. Since participants in this study were high-risk populations in areas with limited literacy, the 24-hour dietary recall was selected for assessment.

In the reliability studies, results showed that the median intakes of all nutrient and food groups (except fruit) assessed with FFQ2 were higher or equal to the median intakes of all nutrients and food groups assessed with FFQ1. It could be that participants were more likely to pay attention to their dietary intakes after the first FFQ, or it could be that over a longer span of time, the dietary of participants changed. The ICCs of most food groups and nutrients ranged from 0.53 to 0.73. After energy adjustment, most ICCs are 0.49 to 0.73. Other reliability studies have reported similar ICC [30–34]. When the food group and nutrient intake were divided into quartiles, the effective quartile coincidence rate between two FFQs was high (ranged from 72.2 to 85.9%). It is rare for all nutrient and food groups to be misplaced in the extreme quartile. Most of the weighted k values have a moderate degree of conformity, only vegetables showed poor consistency. The percentage of participants correctly classified into the same or adjacent categories was slightly higher than reported in other confirmatory studies, while the weighted K values were similar to those reported in other studies [35–37]. In addition, our study collected FFQ data on household condiments commonly used, which made this dietary data more comprehensive than those reported in other studies [24]. In terms of results, FFQ showed acceptable reliability among participants.

The correlation coefficient of validity was less than that of reliability. It is possible that some foods were not consumed regularly and were not recorded in the 24-hour dietary recalls. The median intakes for most of the FFQ were higher than the 24-hour dietary recalls. This finding is consistent with other studies [38–40] that showed FFQ was overestimated. The study also found that the energy adjustment reduced the correlation coefficients of most nutrients, possibly because the nutrients intakes and types of foods varied from person to person. These results are similar to those of other studies [41–43]. The Bland-Altman method was used to evaluate the consistency between the two methods graphically. The results showed that the two methods were comparable, although the average of the differences suggested that FFQ slightly overestimated some nutrients.

It had to be admitted that this study has some limitations. As with all dietary assessment measures, FFQ and 24-hour dietary recall rely on self-reported data, which may be subject to some reporting bias [37]. On the other hand, only two 24-hour dietary recalls were conducted on a random day as there was no significant difference between weekdays and weekends in the diets of residents in areas in this study, and if the number is increased, the consistency between the dietary recall and FFQ will be more authentic and reliable. In addition, this study only used the 24-hour dietary recall for validity study, and the results would be more convincing if biomarkers were used.

## Conclusions

In summary, our SFFQ showed acceptable reliability and reasonable validity in assessing food groups and nutrients intakes for the population at high risk for CVD. Based on this study, the SFFQ is suitable for the assessment of dietary intake of people at high risk of CVD in Ningxia, China.

## Supplementary Information

**Additional file 1.** Annex.

## Data Availability

The datasets used and/or analyzed during the study are available on request from the corresponding authors.

## Abbreviations

CVD: Cardiovascular and cerebrovascular diseases
24HDR: 24-hour dietary recall
FFQ: Food frequency questionnaires
SFFQ: Semi-quantitative food frequency questionnaire
BMI: Body mass index
ICC: Inter-class correlation coefficient
SD: Standard deviation
WHR: Waist-to-hip ratio
LOA: Limits of Agreement
