# Supplementary figures and images for: Validity and reliability of a semi-quantitative food frequency questionnaire in groups at high risk for cardiovascular diseases
Source: Nutr J. 2022 Oct 14;21:63. doi: 10.1186/s12937-022-00815-8 (PMC9569079; doi:10.1186/s12937-022-00815-8)

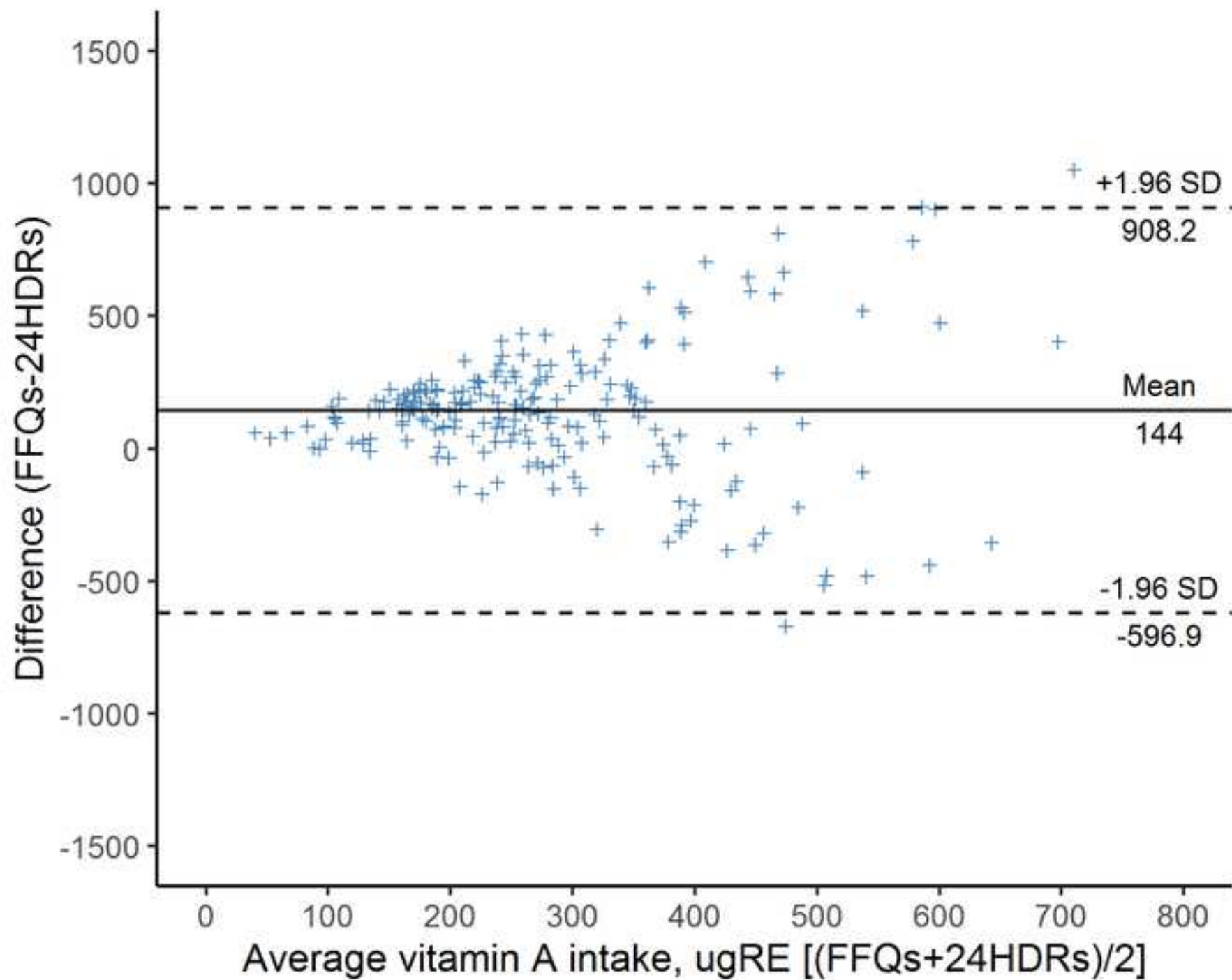

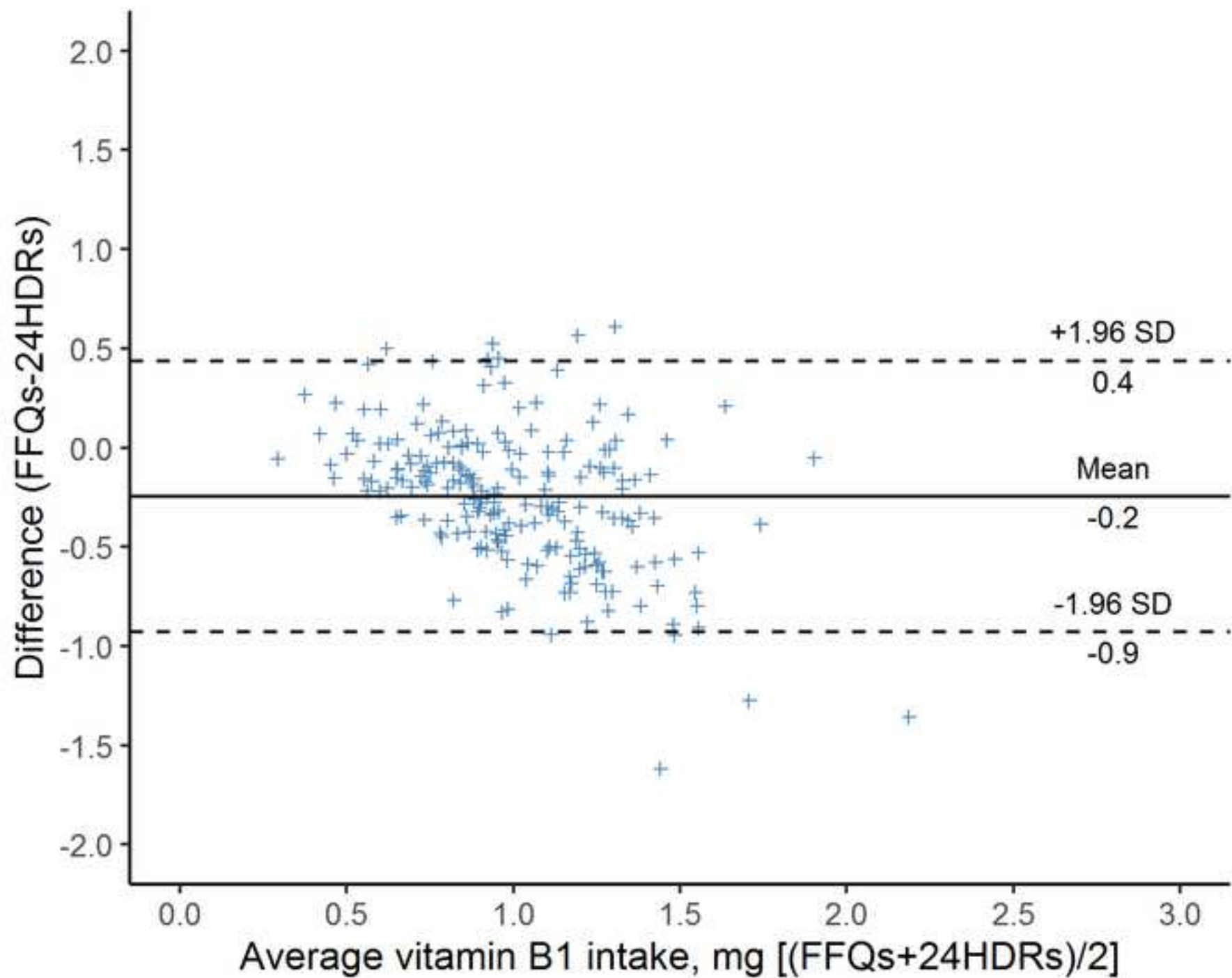

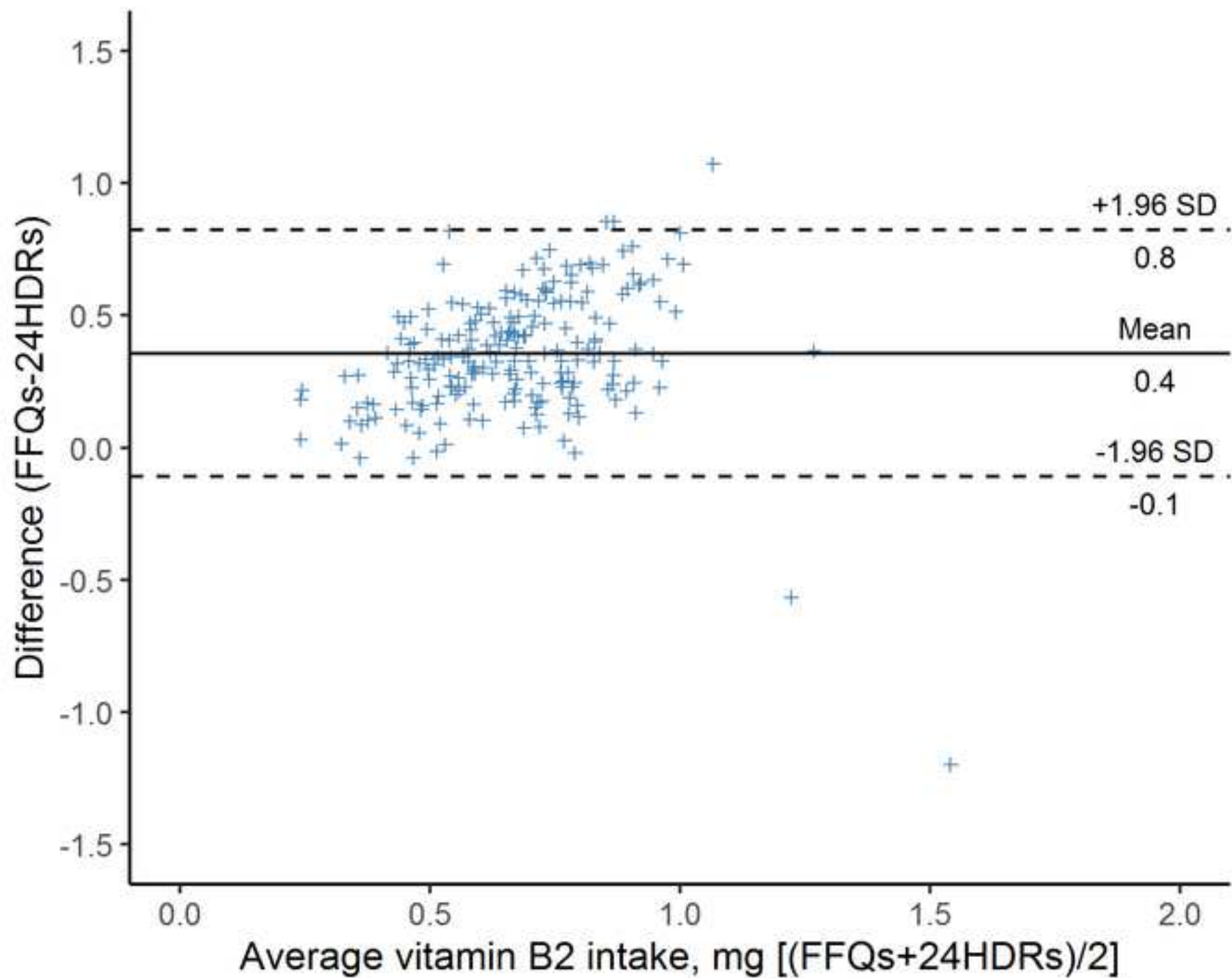

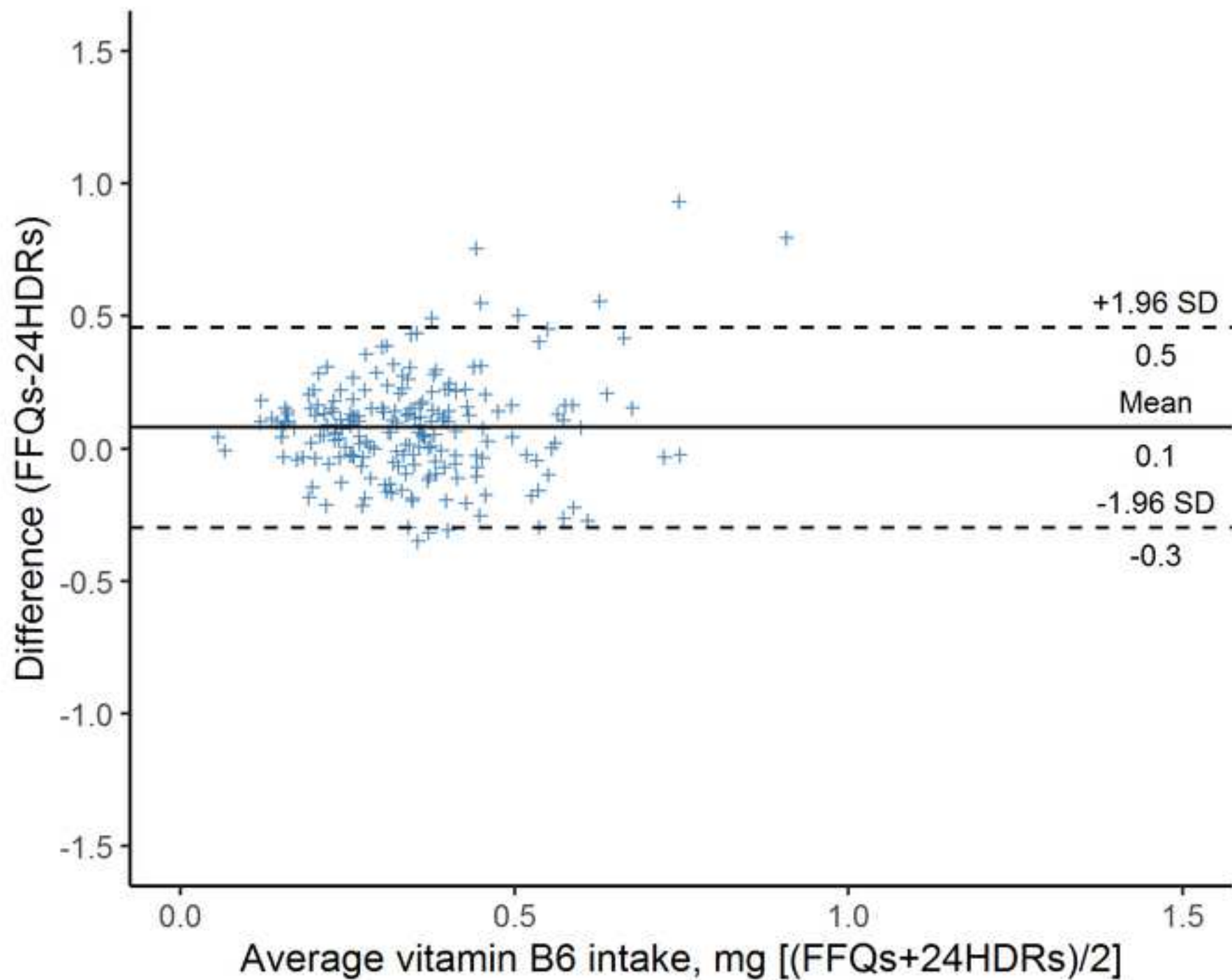

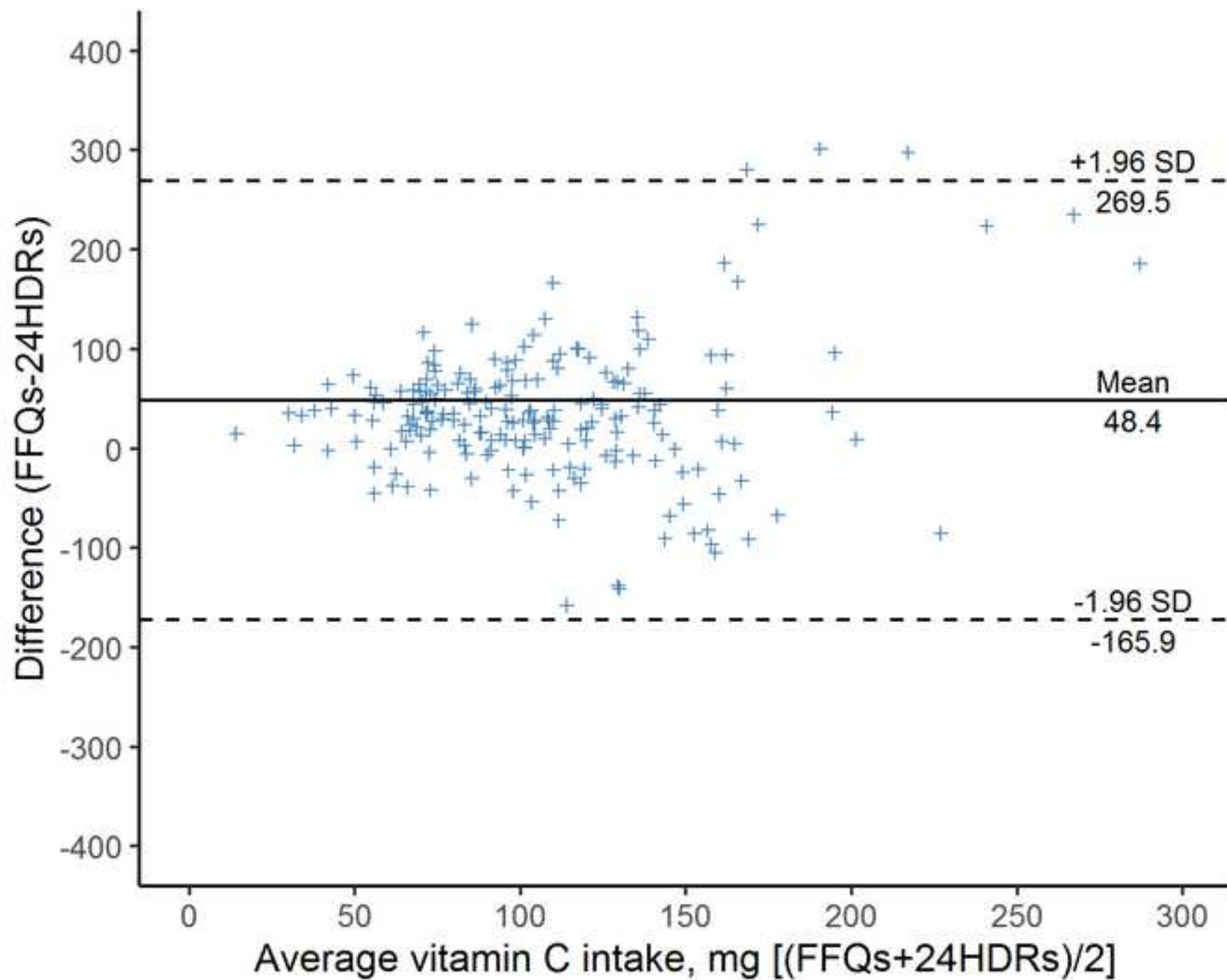

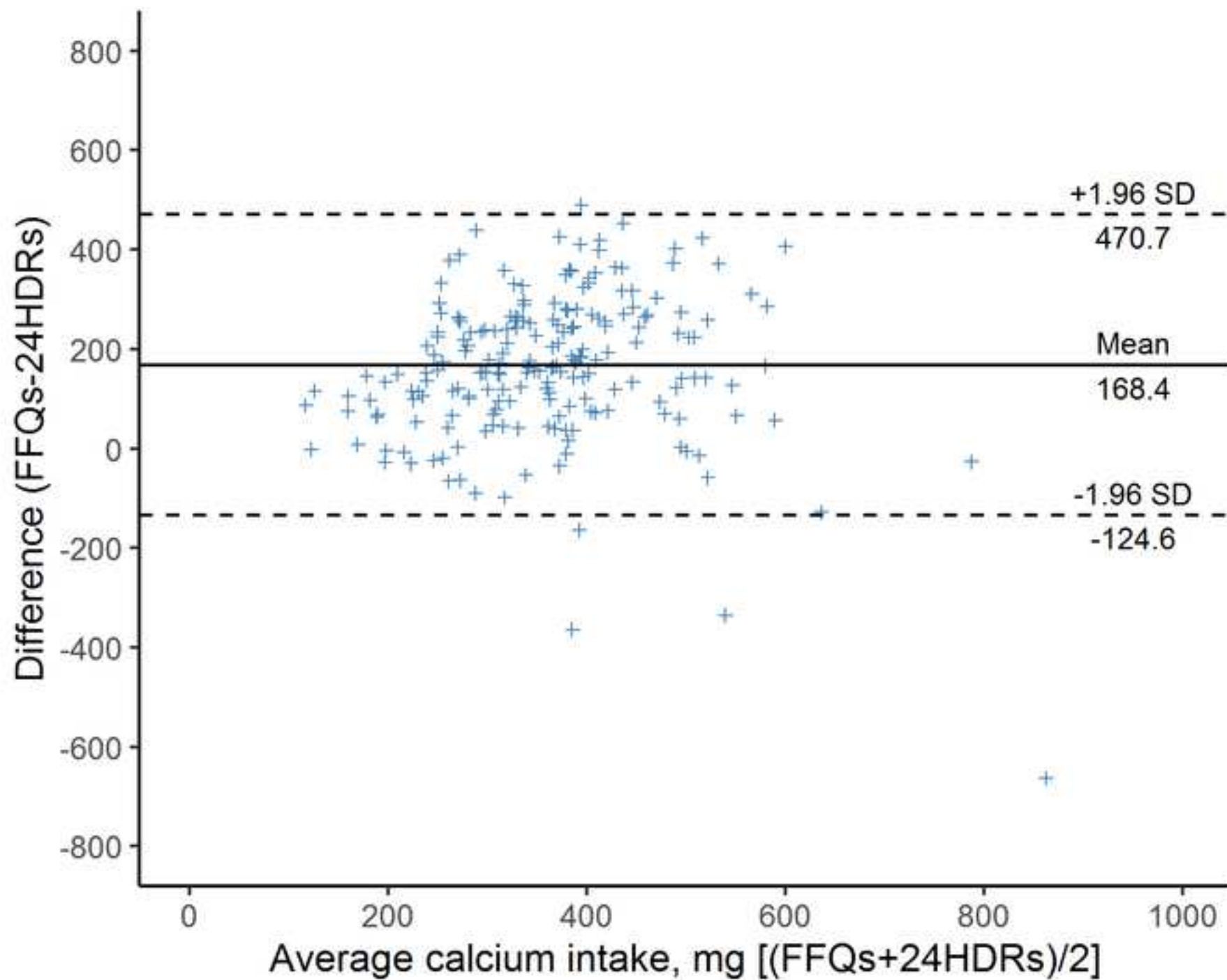

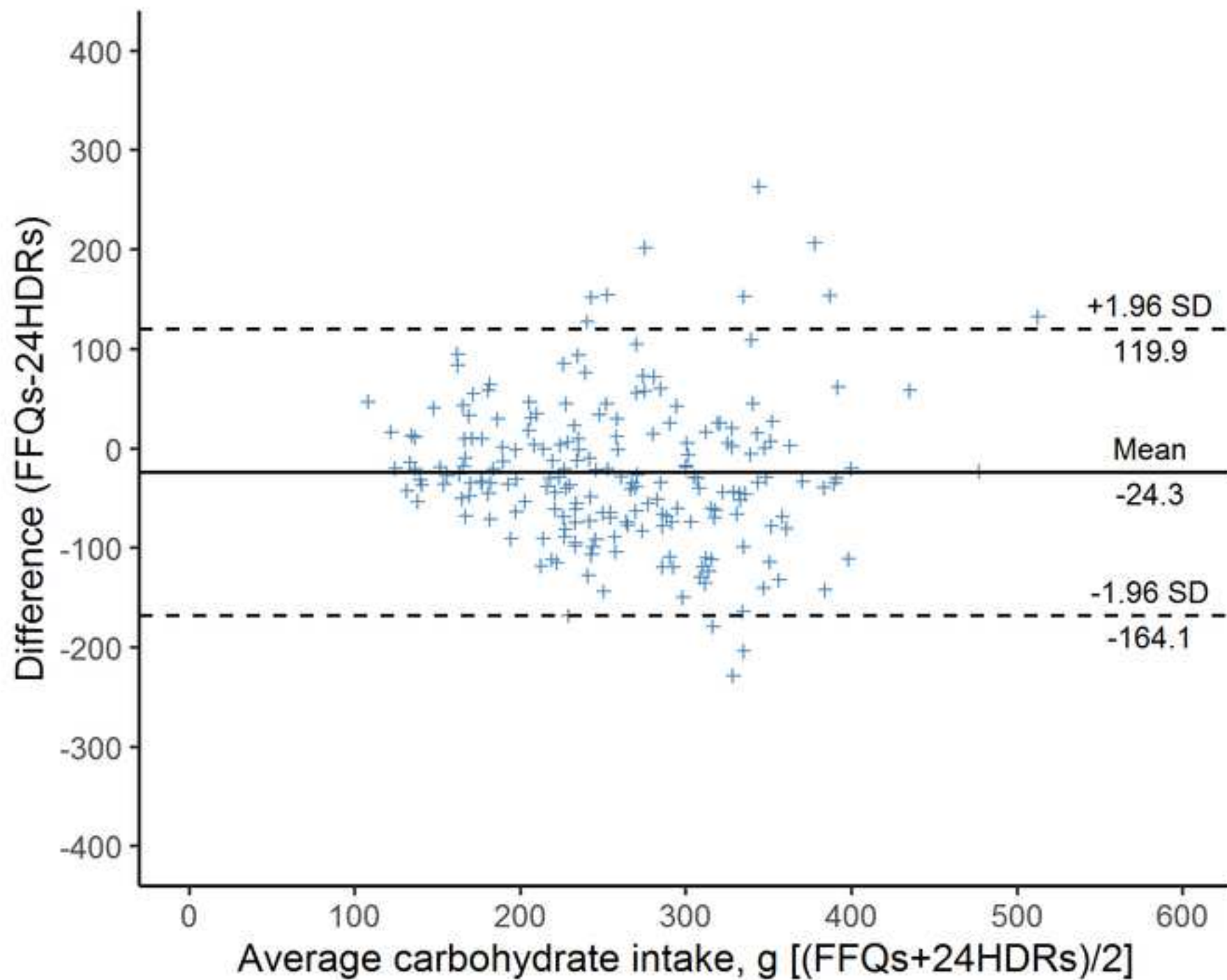

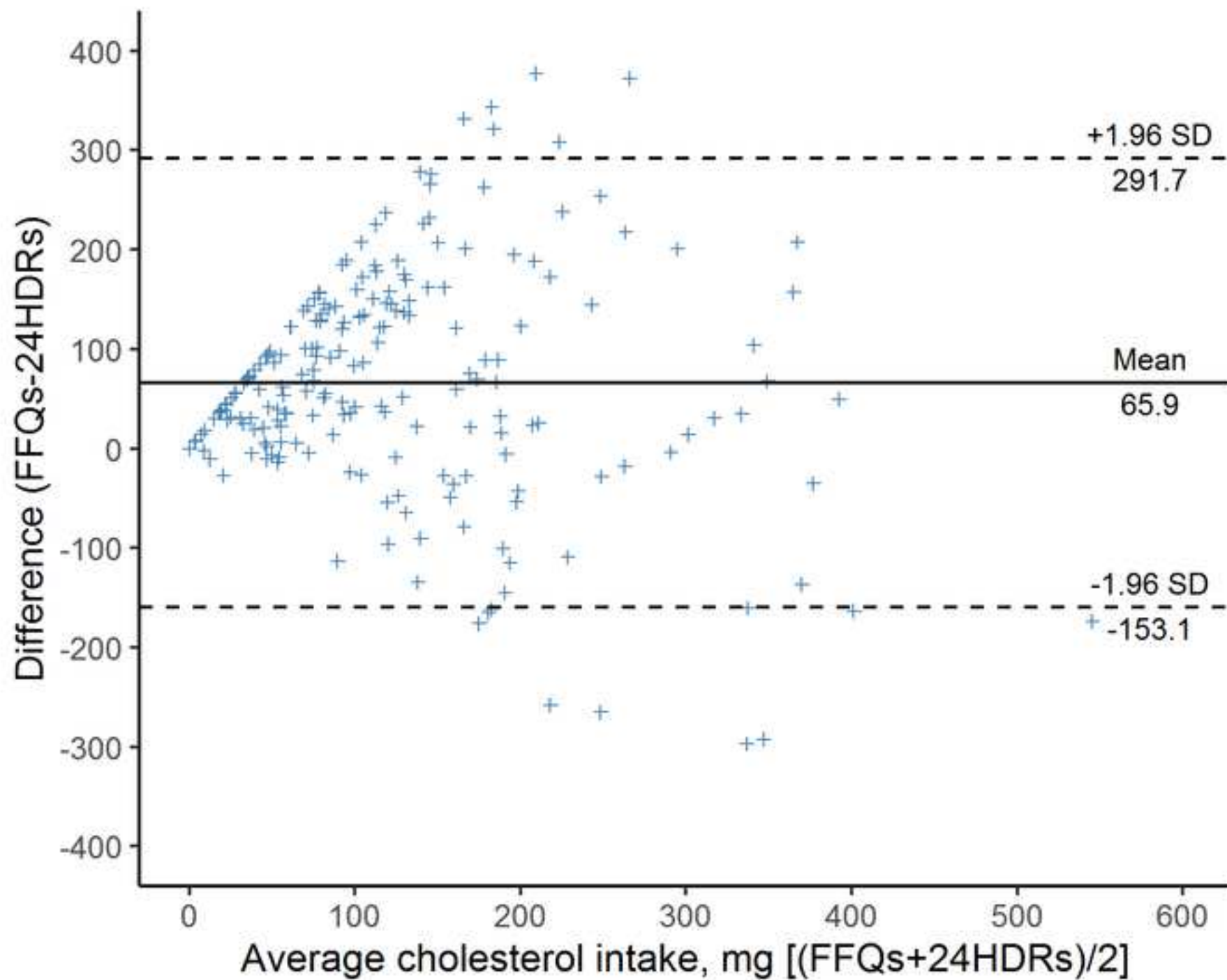

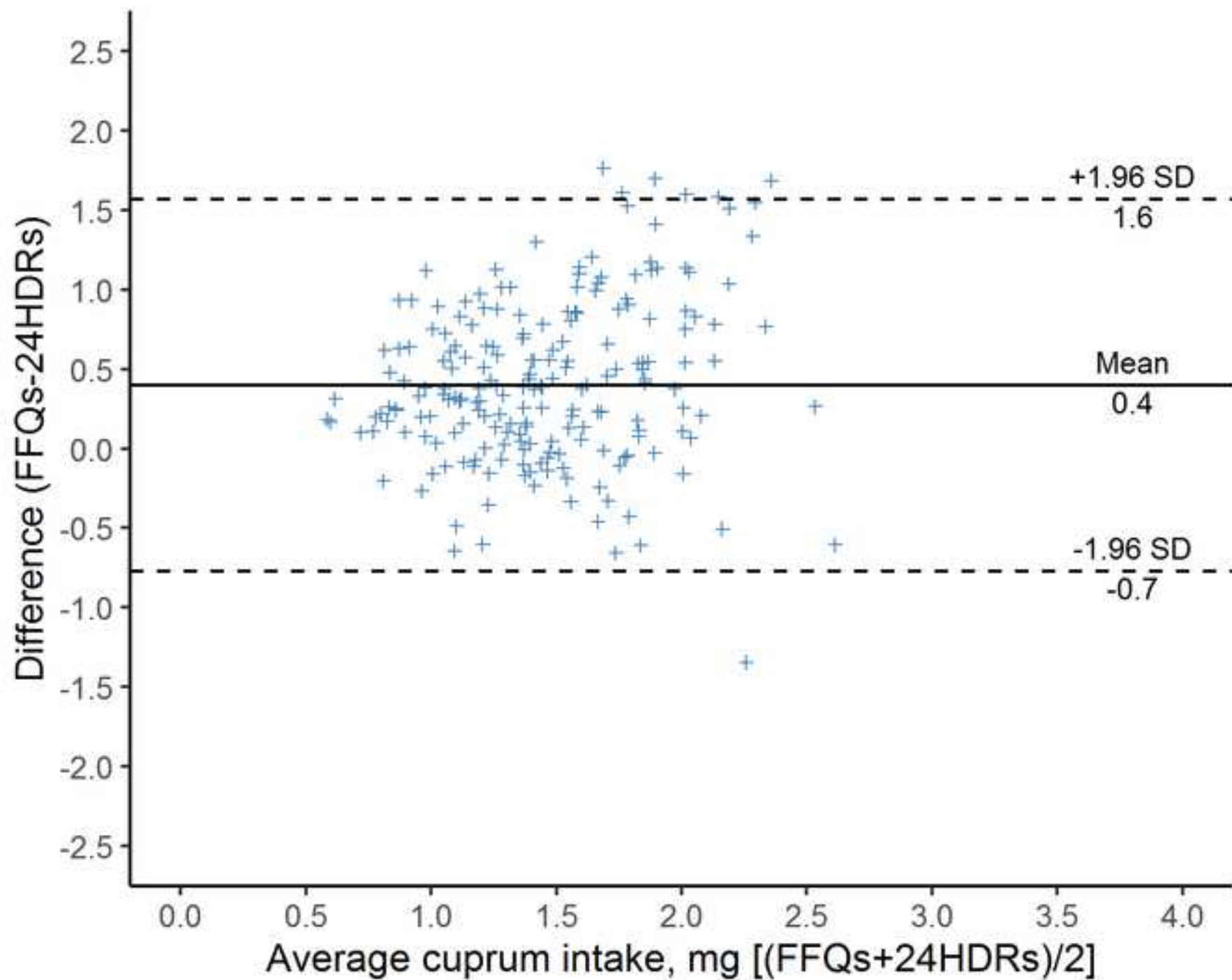

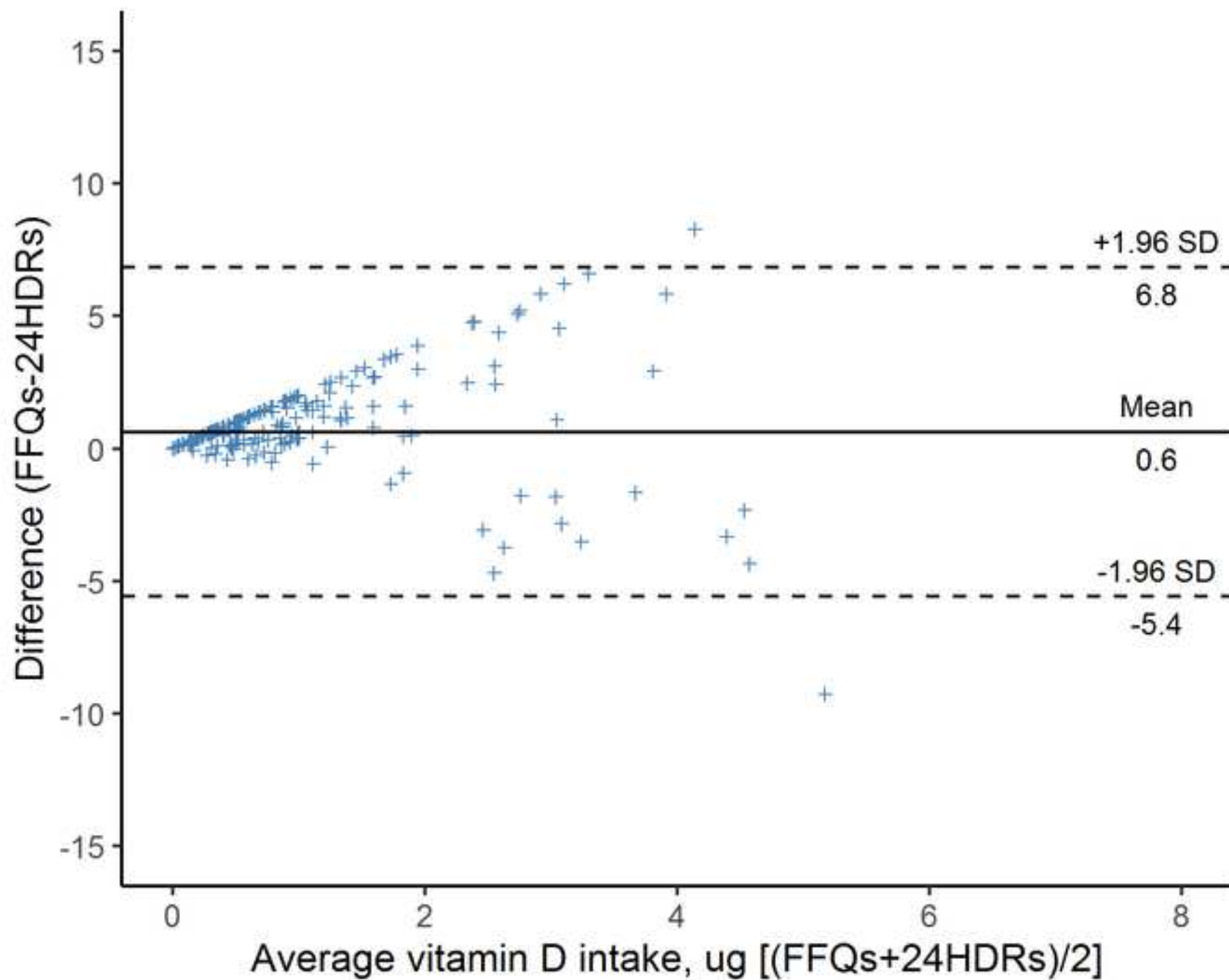

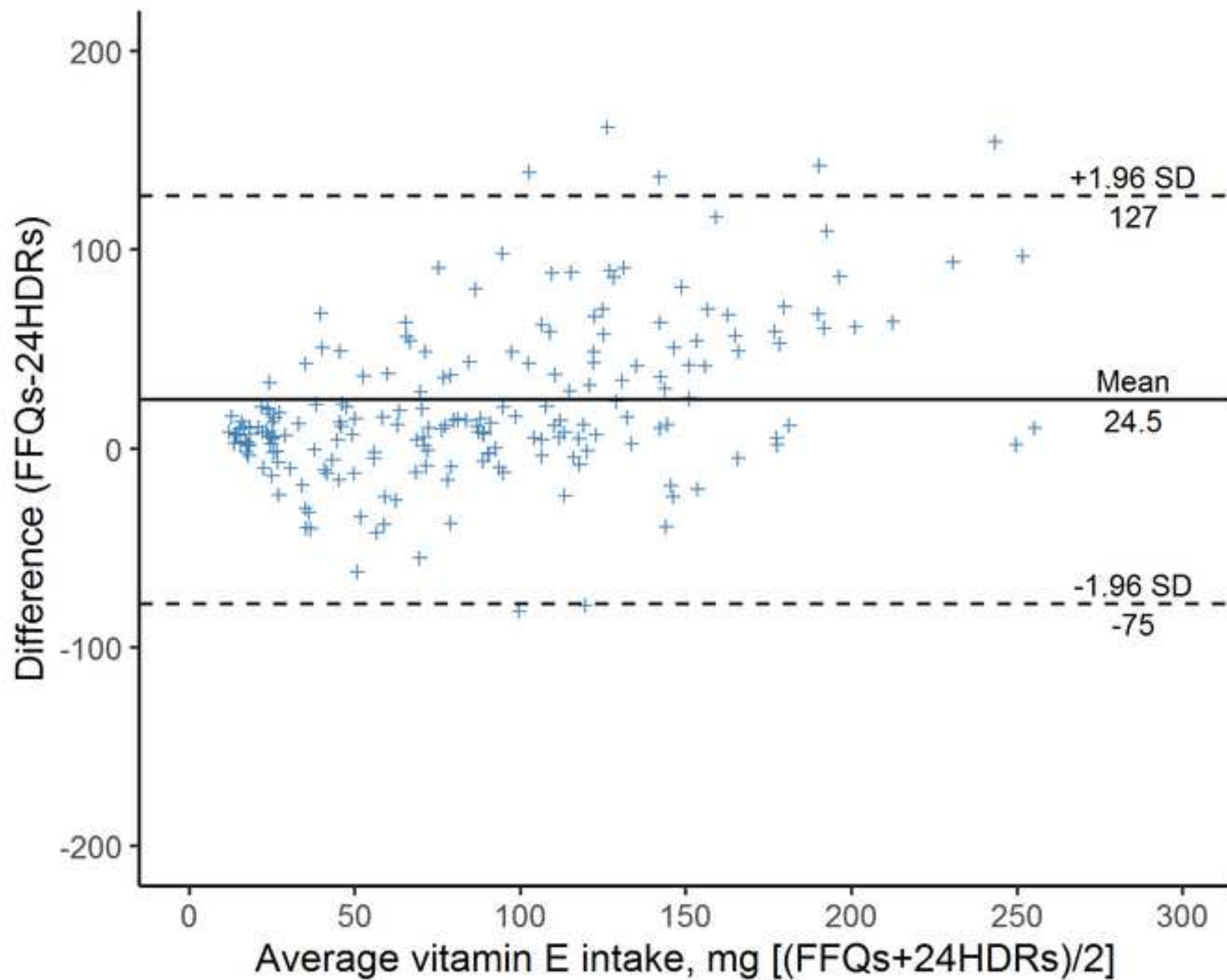

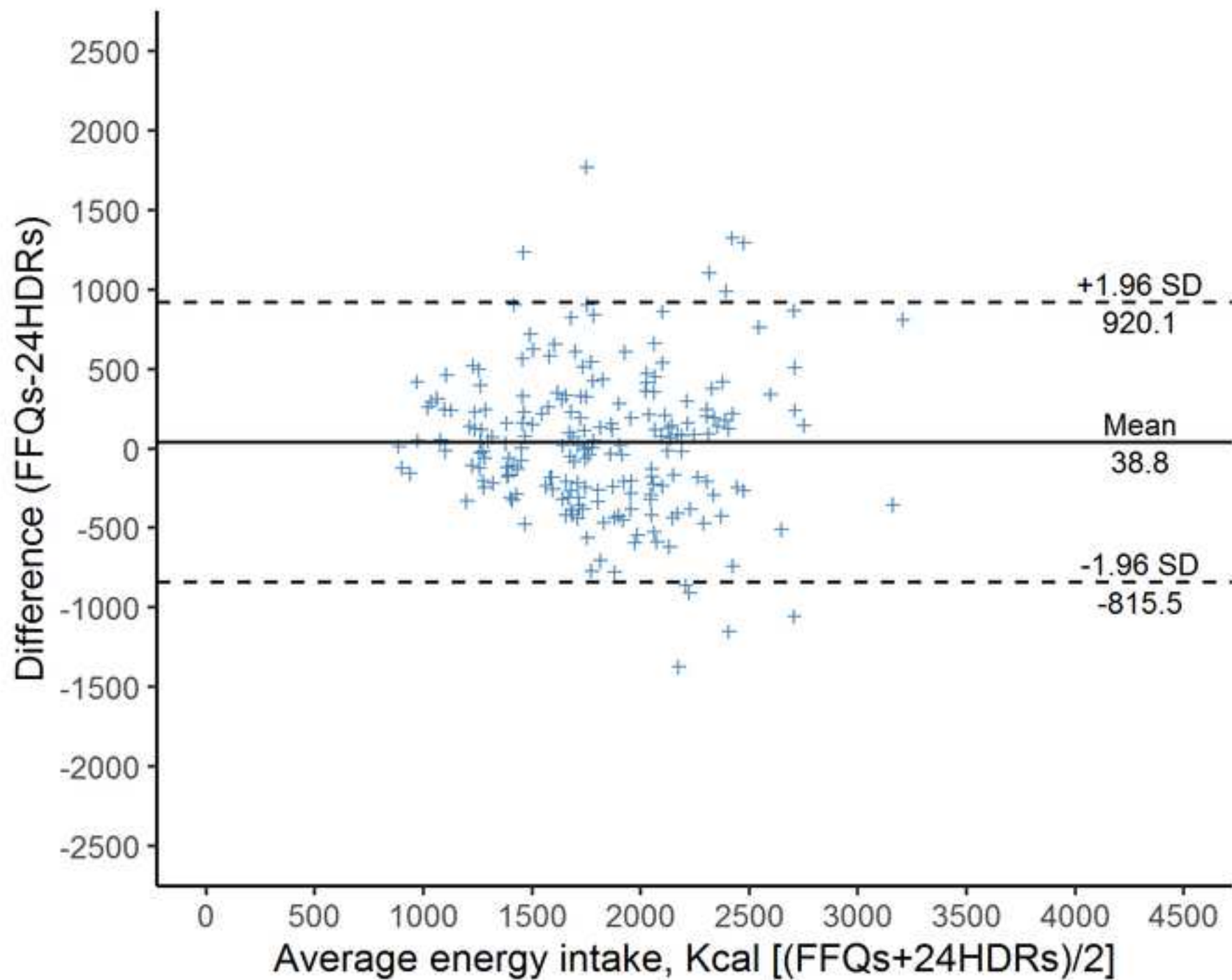

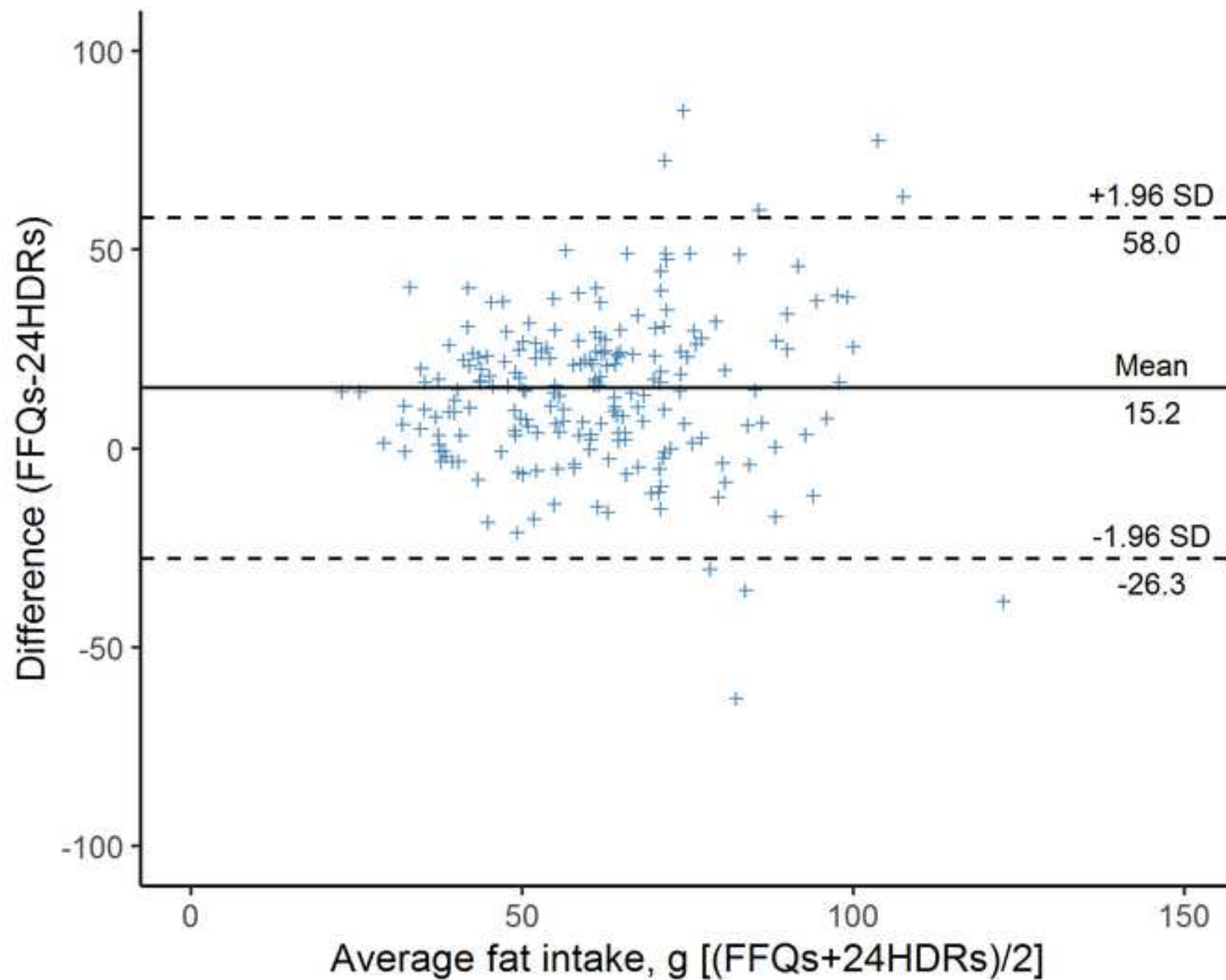

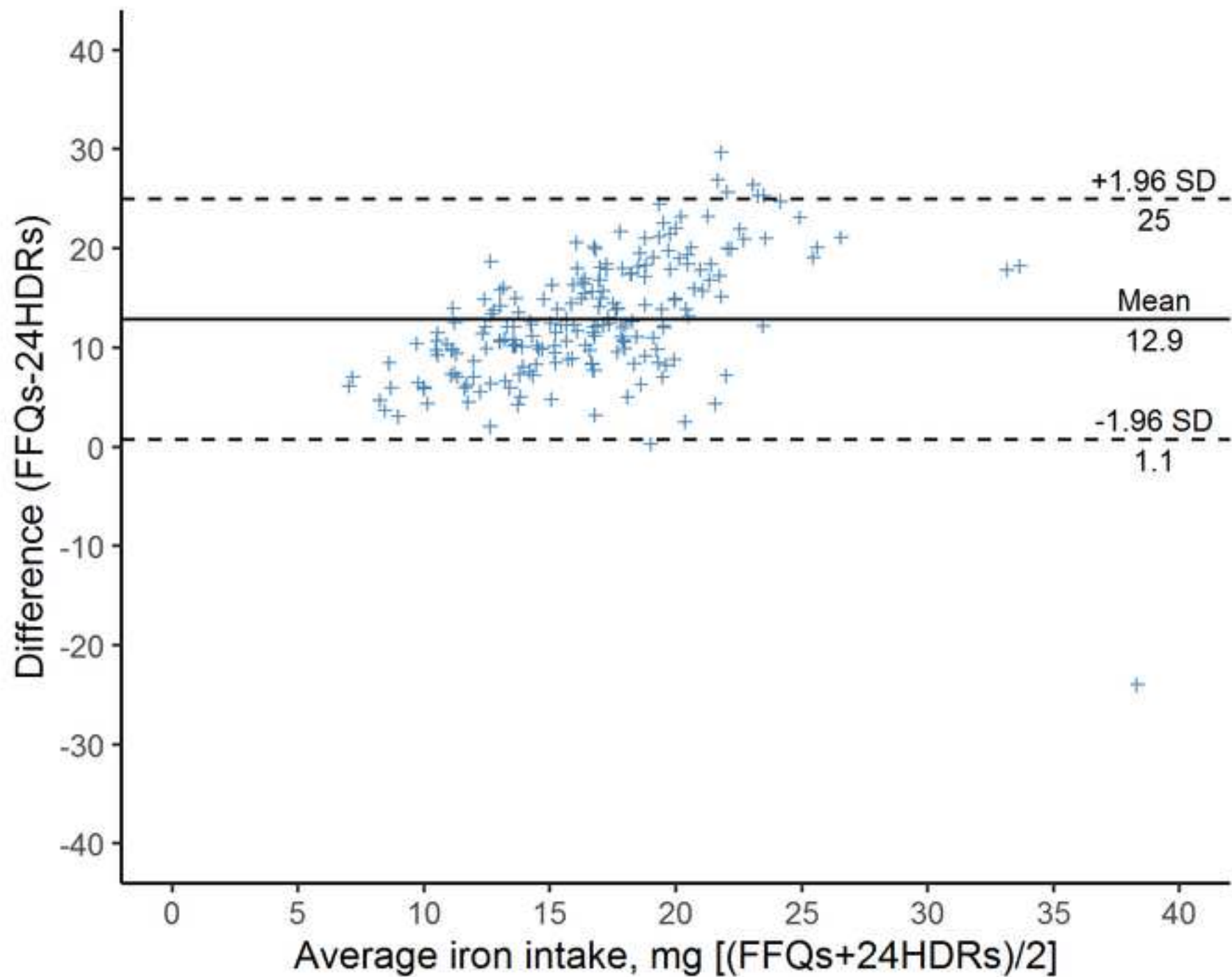

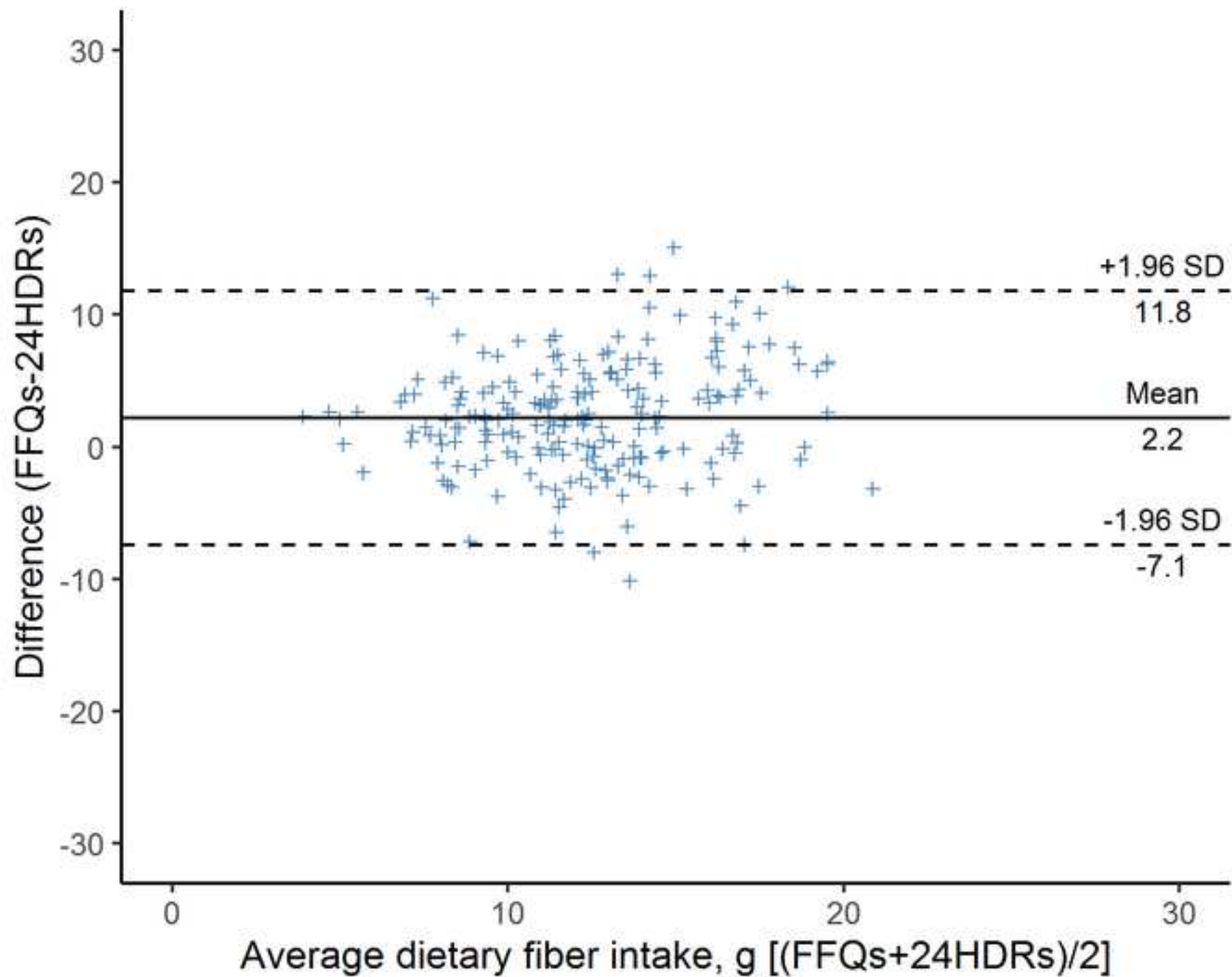

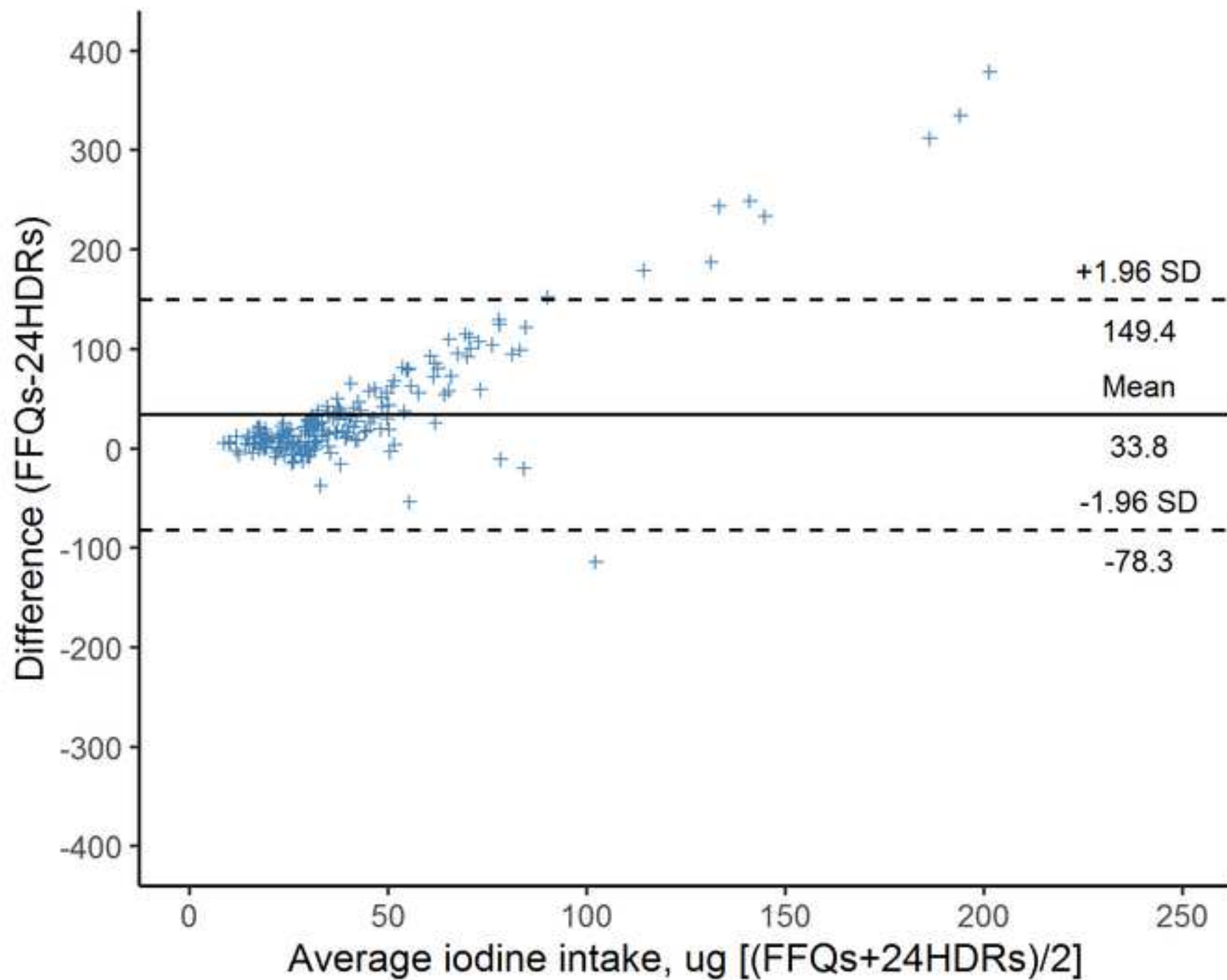

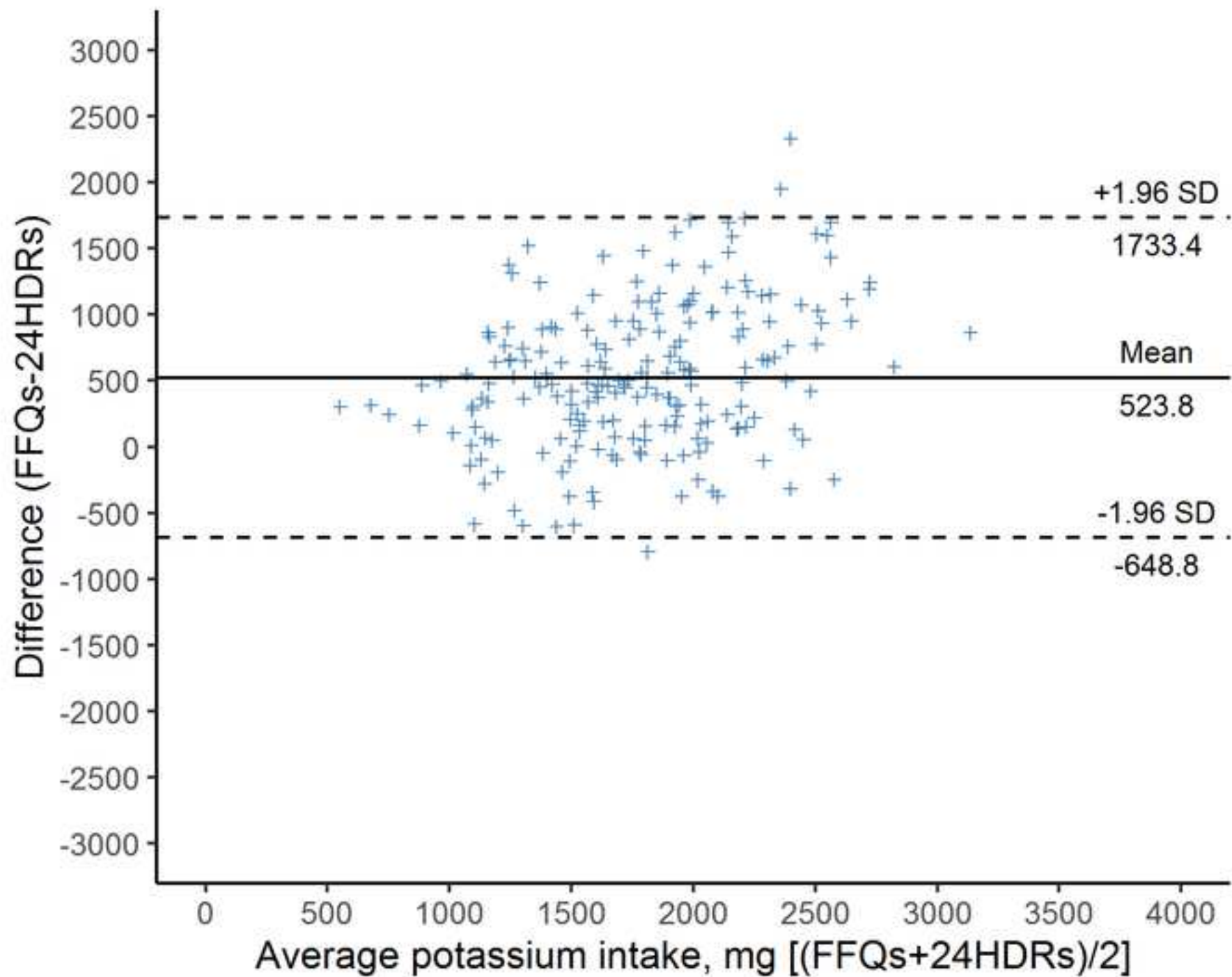

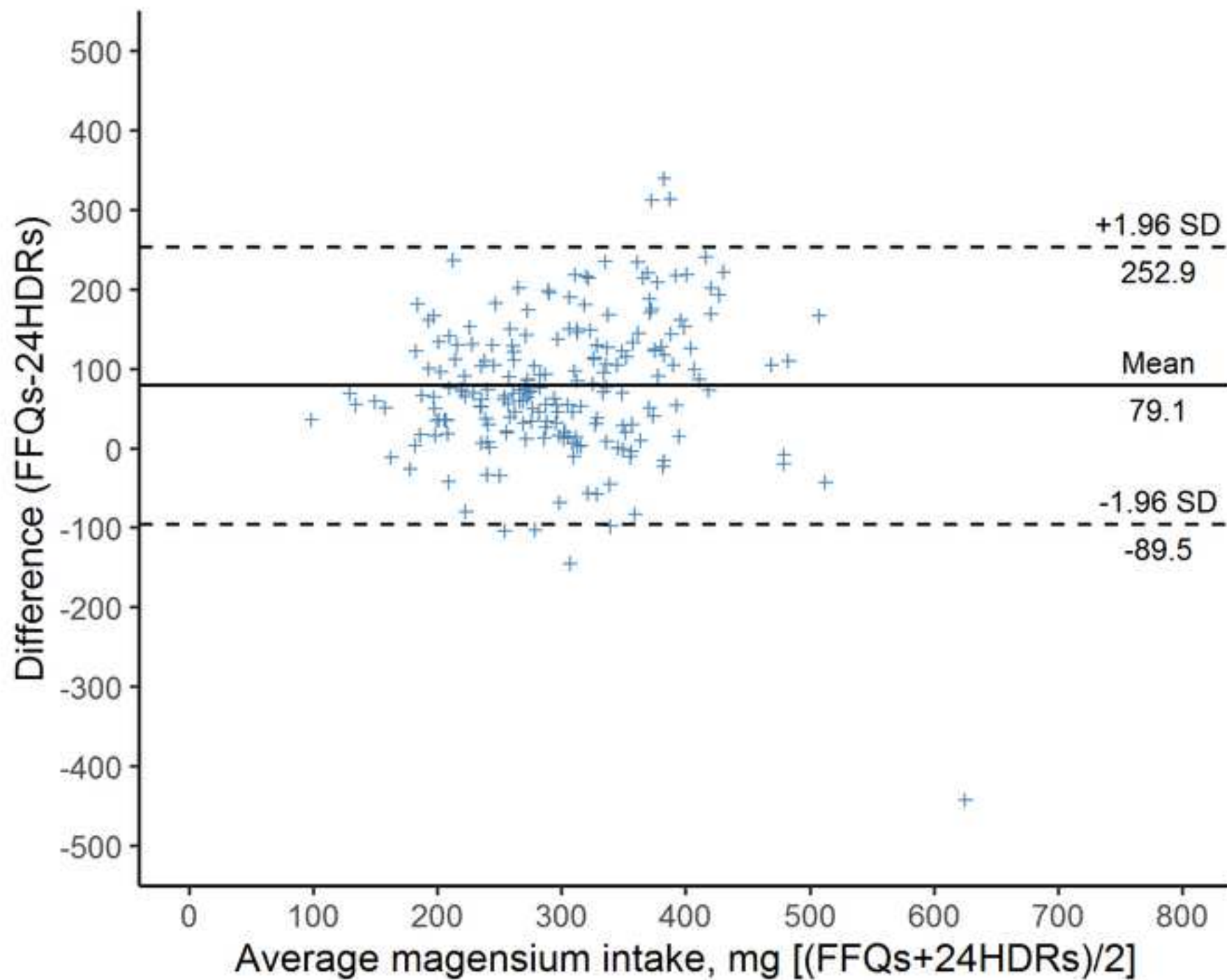

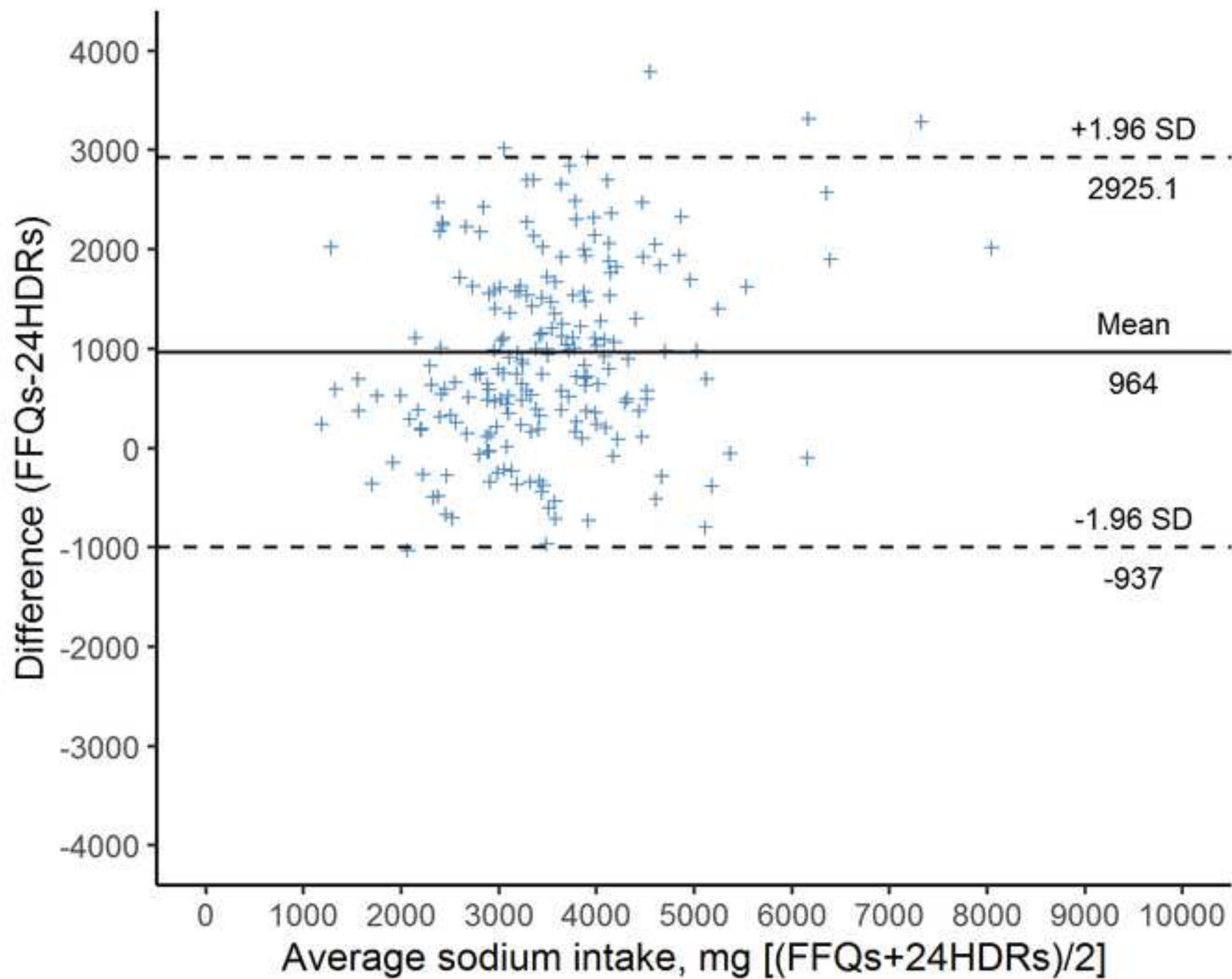

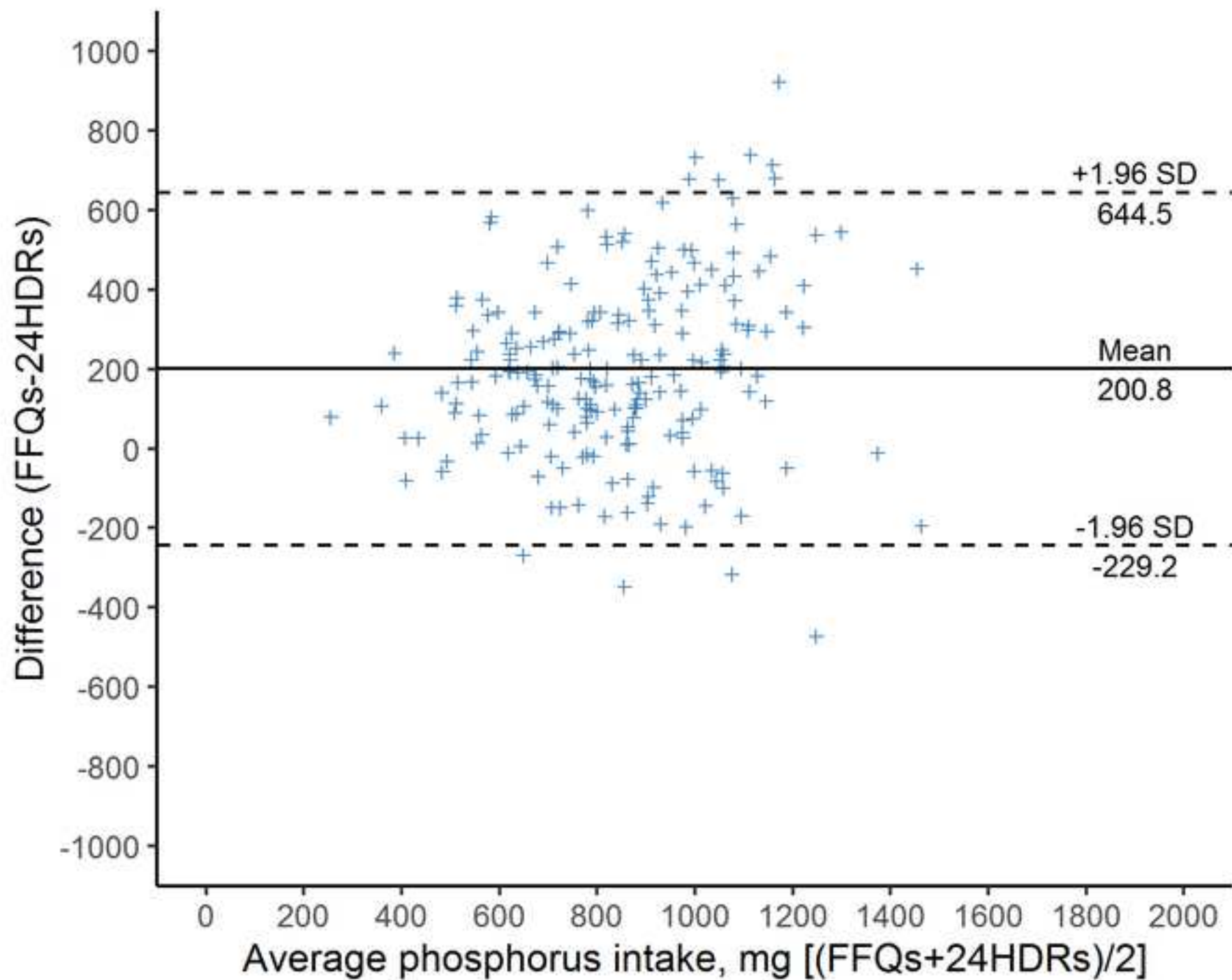

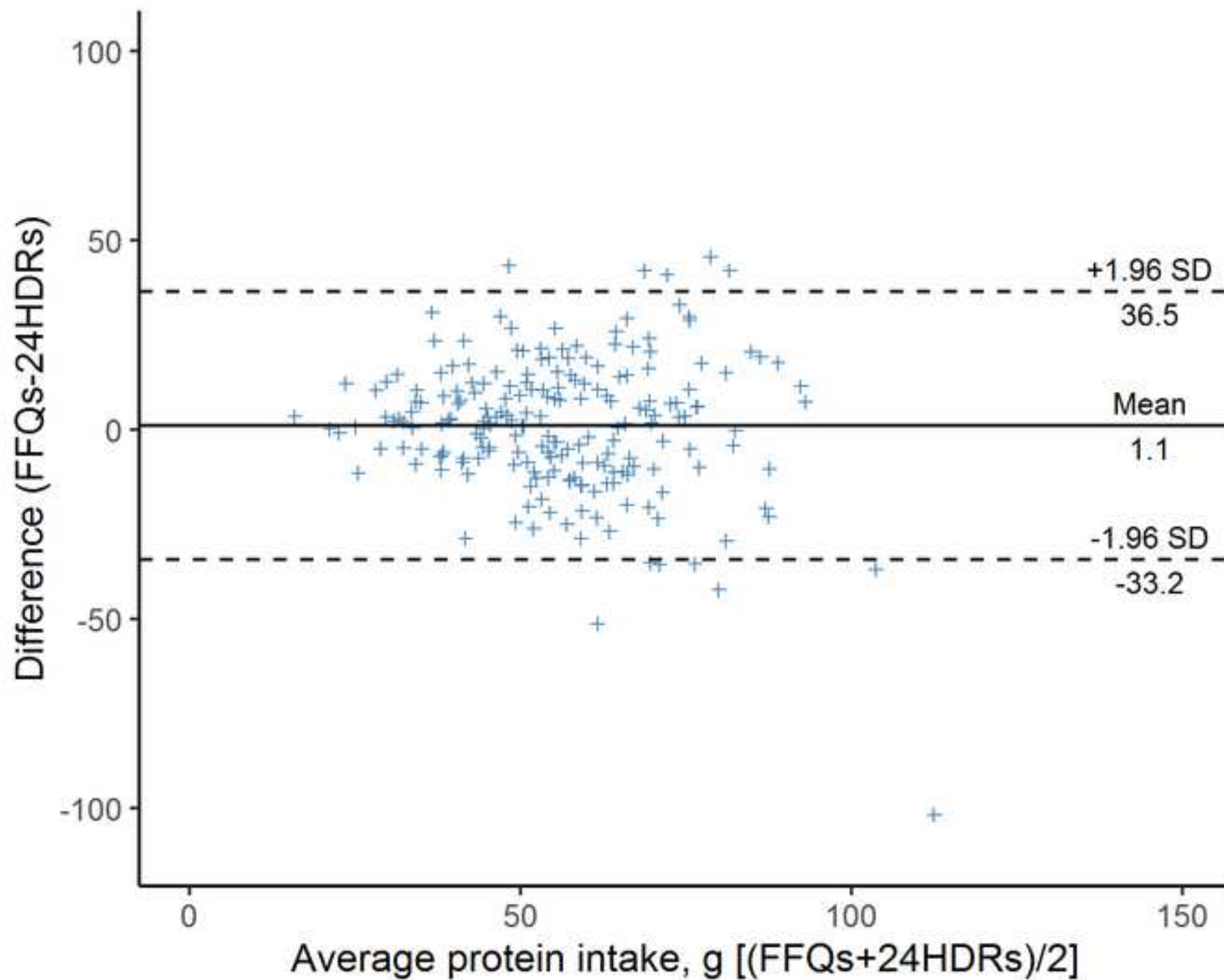

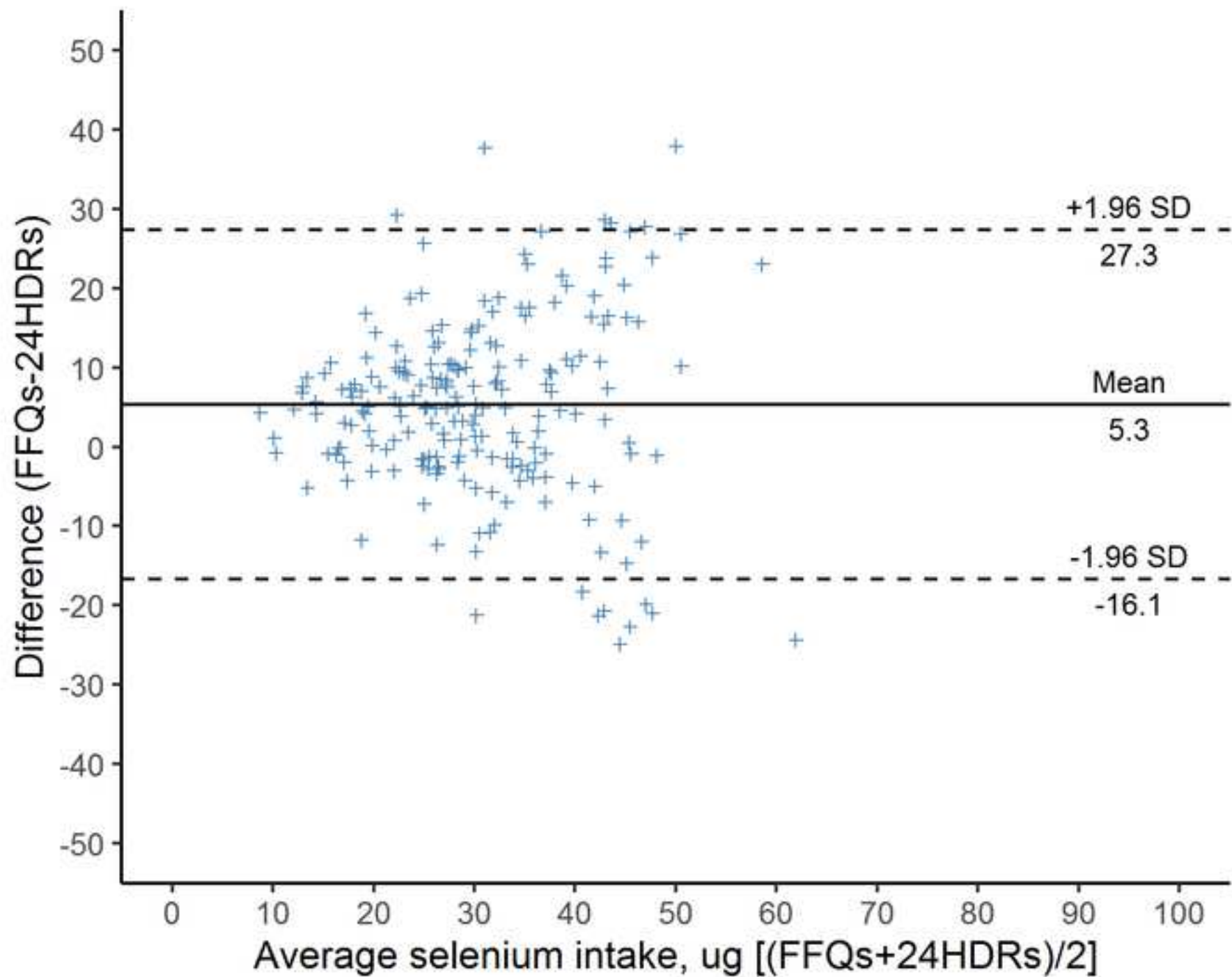

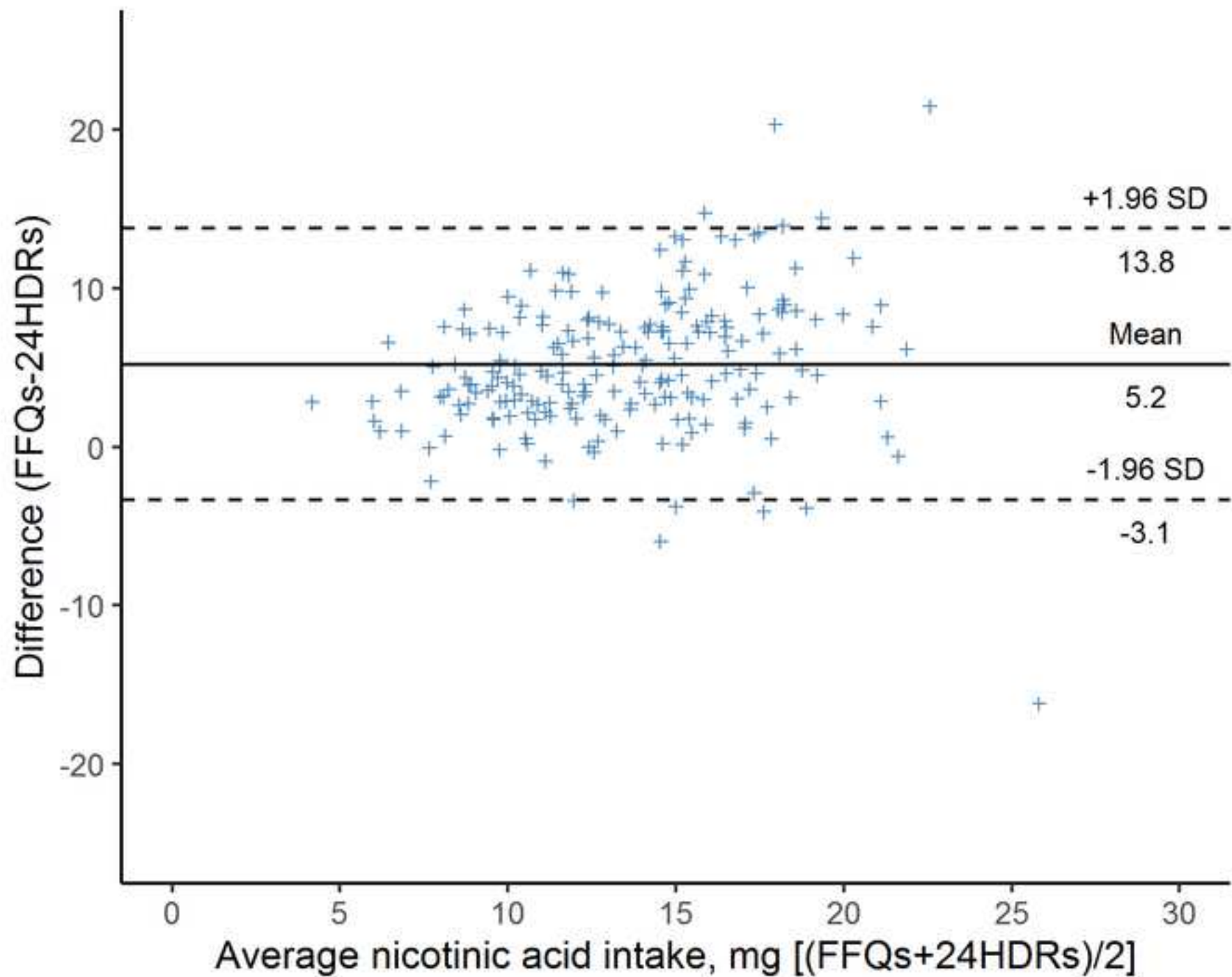

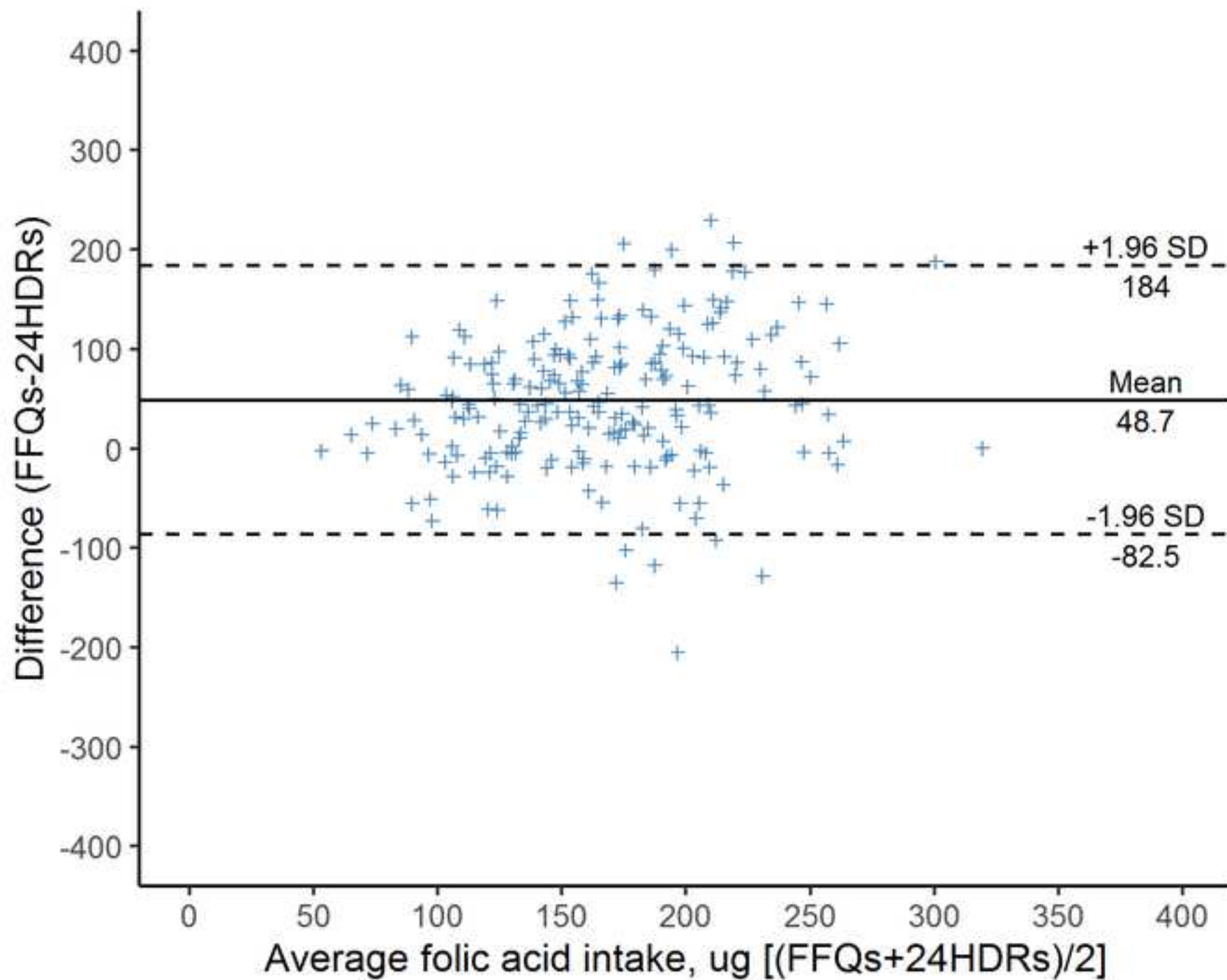

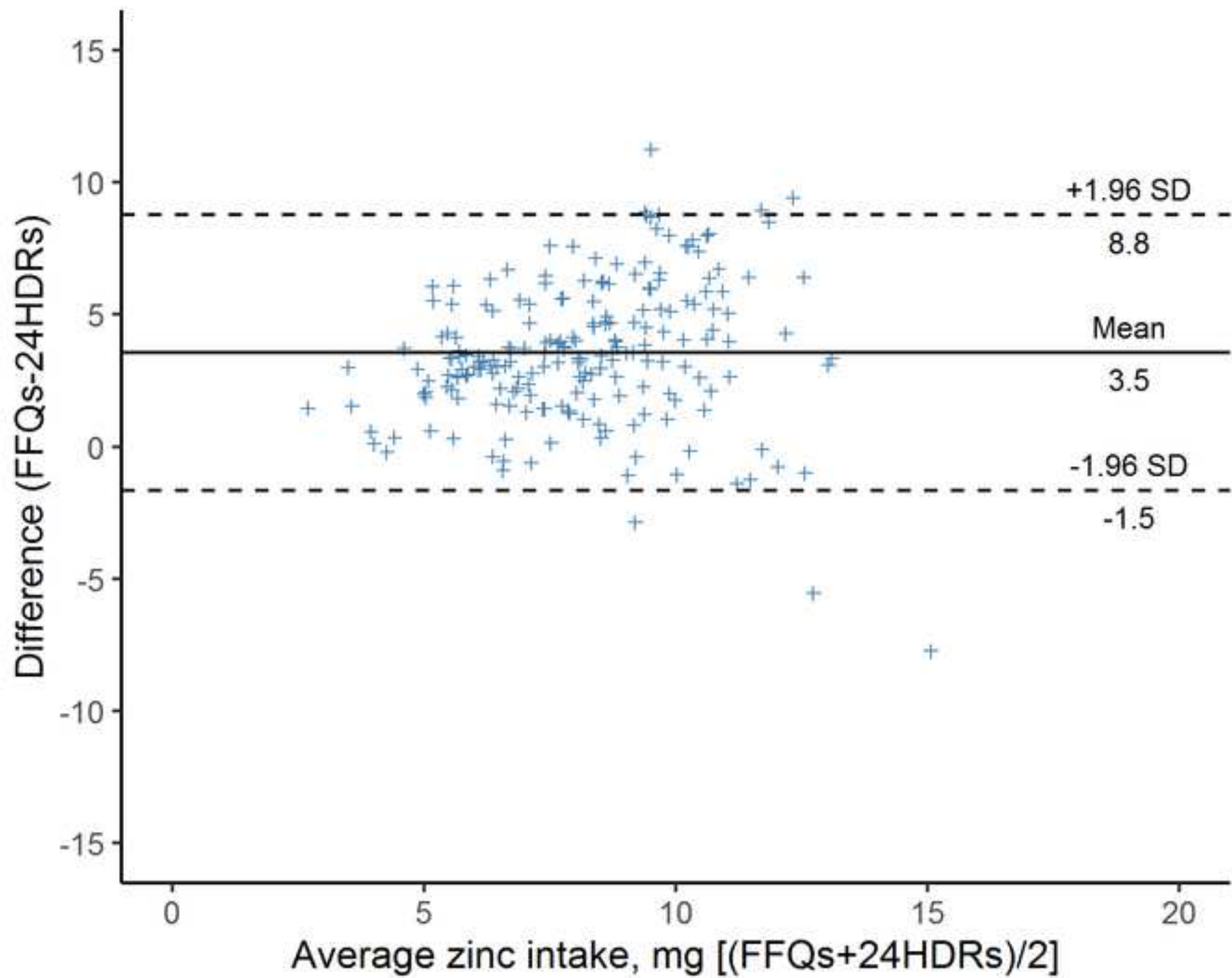

Supplement: Supplementary file 1 — Additional file 1. Annex. [file 12937_2022_815_MOESM1_ESM.pdf]
